# Apolipoprotein L1 (APOL1): Consideration of Molecular Evolution, Interaction with APOL3, and Impact of Splice Isoforms Advances Understanding of Cellular and Molecular Mechanisms of Cell Injury

**DOI:** 10.3390/cells14131011

**Published:** 2025-07-02

**Authors:** Razi Khalaila, Karl Skorecki

**Affiliations:** 1 Department of Nephrology, Rambam Health Care Campus, Haifa 3109601, Israel; karl.skorecki@biu.ac.il; 2 Departments of Genetics and Developmental Biology, Rappaport Faculty of Medicine and Research Institute, Technion-Israel Institute of Technology, Haifa 319060, Israel; 3 Azrieli Faculty of Medicine, Bar-Ilan University, Safed 1311502, Israel

**Keywords:** trypanosomiasis, trypanosome lytic factor, APOL1 risk variants, APOL1 splice variants, APOL3, signal peptide, phylogenetics, protein–protein interaction

## Abstract

The Apolipoprotein L1 (APOL1) innate immunity gene product represents the sole member of the APOL gene family in humans capable of secretion into circulation, thereby mediating the trypanolysis of *T. brucei brucei*. Gain-of-function variants of the APOL1 gene originated and spread among human population groups to extend APOL1’s protective capacity to include also serum-resistant subspecies, such as *T. brucei gambiense* (S342G known as APOL1-G1) and *T. brucei rhodesiense* (N388_Y389del known as APOL1-G2). The biochemical pathways underlying the lytic activity of these evolutionary favored mutations against bloodstream trypanosomes have been elucidated with remarkable precision. However, the intricate molecular mechanisms by which such variants confer an increased susceptibility to renal cellular injury and consequent kidney disease remain incompletely defined. In the absence of a consistent mechanistic explanation for differential kidney injury, we propose pursuing three interrelated avenues of investigation informed by prior epidemiological and mechanistic evidence linking them to APOL1’s cytotoxicity: (1) Molecular evolution of APOL1 haplotypes in human populations, (2) APOL1 splicing and consequent splice isoforms, (3) Interaction of APOL1 with other APOL gene family members, prioritizing APOL3. In the current study, we use reanalysis of population genetics datasets to resolve the haplotype contexts of all protein-altering APOL1 variants, uncovering previously unrecognized variant–haplotype couplings. We further characterize distinct cellular physiological properties among APOL1 splice isoforms, stressing the importance of isoform vB and what can be learned from isoform vC. Finally, a native interaction, and its interface, between APOL1 and APOL3 is reported, and shown to be differentially modulated by G1 and G2. We contend that continuing studies integrating these three interrelated domains will substantially advance mechanistic insights into APOL1 variant-driven renal injury, and leverage the findings to provide a more cohesive framework to guide future research.

## 1. Introduction

It was a massive outbreak of African trypanosomiasis (sleeping sickness), or *nagana* in the native tongue, in the then British-controlled Zululand, that lead to the discovery of the causative agent and the mode of transmission of the disease by David Bruce in 1896. Bruce demonstrated that sleeping sickness is essentially a tsetse fly disease, caused by protozoan parasites transmitted by infected tsetse flies to livestock and humans. Named after Bruce, two subspecies of *Trypanosoma brucei* were distinguished, *T. brucei brucei* as “essentially a parasite of animals”, since it was non-infectious to humans, and *T. brucei gambiense* as “a parasite of man” [1]. Soon after, another *T. brucei* subspecies was discovered to be pathogenic in non-African travelers in Zimbabwe (then called Rhodesia) and named *T. brucei rhodesiense* [2]. This differential capacity of *T. b.* subspecies to infect humans was explained a few decades later by the presence of a human serum trypanolytic factor, to which only *T. b. brucei* was susceptible, in contrast to *T. b. rhodesiense*, which was resistant [3].

The identity of the molecule responsible for the lysis of *T. b. brucei*, eventually termed trypanosome lytic factor (TLF), was revealed to reside in high density lipoprotein (HDL) [4], with subsequent further refinement to a minor subclass of ‘cytotoxic’ HDL particles with unusually large size (~500 kDa), comprising a number of unique proteins including Apolipoprotein AI (ApoAI) [5]. This notion snowballed into prominence in the scientific mainstream until a different TLF was discovered, being almost double the size but devoid of any HDLs, and with the same trypanocidal activity [6]. This discrepancy was subsequently resolved when the existence of two TLFs was confirmed: the TLF molecule that co-purifies with the HDL fraction, termed now TLF1, was shown to be completely inhibited by exogenous haptoglobin (Hp) at concentrations similar to those in human serum, and its ‘aberrant’ trypanocidal activity observed in in vitro cultures was only possible with the removal of Hp. In contrast, the larger TLF entity, dubbed TLF2, was unaffected by the addition of haptoglobin and exerted the same trypanocidal activity as normal human serum [7]. Although ApoAI and haptoglobin-related protein (Hpr), a hemoglobin binding protein, were found to be major components of both TLFs [8], IgM was unique to TLF2, and further characterization of TLF1 and TLF2 trypanocidal activity provided incontrovertible proof that TLF2 indeed accounts for most of the trypanolytic activity in human serum [9]. The use of monoclonal antibodies against ApoAI and Hpr was sufficient to stave off trypanolysis by both TLFs, and to lend credence to the conclusion that those proteins are the active trypanolytic agents in TLFs [10].

In parallel, HDL particles provoked an unremitting attraction due to their pivotal role in cholesterol metabolism, and their possible involvement in numerous human pathologies, including cardiovascular diseases and preeclampsia [11]. This culminated in the discovery of a novel protein constituent of HDL particles, named Apolipoprotein L (ApoL), that existed in two forms in circulation, a 42-kDa and a 39-kDa species [12]. Further genomic analysis of the ApoL locus revealed that the newly discovered ApoL protein, now termed *APOL1*, was a member of a gene family composed of five additional genes, grouped within 619 kb on human chromosome 22 and termed almost sequentially *APOL1-6* [13,14]. The implication of this discovery was far reaching and was eventually brought to its apogee when APOL1 was undoubtedly identified as “the trypanosome lytic factor of human serum” [15]. This landmark work also shed light on APOL1’s mode of action, which involved its uptake by *T. b.* subspecies through the endocytic pathway into their lysosomes, leading to their disruption and ultimately to cell death. This was in accordance with previous reports that implicated endocytosis of TLF in disruption of acidic intracellular vesicles and subsequent killing of trypanosomes [16]. Moreover, the resistance of *T. b. rhodesiense* was finally explained by the expression of a variant surface glycoprotein (VSG)-related molecule termed serum resistance associated protein (SRA) that has evolved the unique function of binding and sequestering APOL1 and thereby inhibiting its trypanolytic activity [17].

The landscape of trypanosome infection and immunity witnessed a seismic shift upon this discovery, and what had ossified into a central dogma for almost two decades was revitalized, thrusting APOL1 into the limelight. Consequently, a number of studies more thoroughly evaluated the trypanolytic activity of APOL1 and the reasons behind engulfing deadly APOL1-contatining particles by trypanosomes. While some suggested a lipoprotein scavenger receptor on the plasma membrane of trypanosomes that binds lipoprotein-rich particles, such as HDLs and TLF1, to supply the lipid auxotroph trypanosomes with much-needed lipoproteins [18], others argued that it was instead a parasite glycoprotein receptor that binds hemoglobin-Hpr complexes to supply heme for growth and resistance to oxidative stress within the host [19]. However, these observations were contested by previous reports of the HDL-free TLF2 being the primary trypanolytic component in human serum, the predominance of Hp-hemoglobin complexes in the serum that would obviate the need to search for alternative sources of heme, and more recent reports demonstrating that, while APOL1 is indeed a component of both TLF1 and TLF2, more than 90% of APOL1 in the human serum is rather enriched in lipid-free large protein complexes [20]. In relation to the core trypanolytic activity of APOL1, the concept that it is through disruption of lysosomes was consolidated by further work delineating several functional domains within its protein sequence, including a membrane pore-forming domain functionally reminiscent of bacterial colicins, through which APOL1 targets lysosomal membranes, forms anion channels within them causing continuous influx of chloride, osmotic swelling and, ultimately, death [21].

Unwittingly, the story of APOL1 came full circle through a seemingly unrelated discovery. It has been long noted that African Americans experience a disproportionate risk of several forms of chronic kidney disease (CKD), including a four-fold increased risk of sporadic focal segmental glomerulosclerosis (FSGS) [22] and an 18- to 50-fold increased risk for HIV-1–associated collapsing FSGS [23] when compared to European Americans. Since this disproportionality was evident in individuals of African descent in other geographic regions, it suggested the possible contribution of a genetic underpinning. Two back-to-back published works using mapping by admixture linkage disequilibrium (MALD) identified variation in a 30MB genomic region, which included the *MYH9* gene as such a contributing factor [24,25]. However, multiple unsuccessful attempts were made to allocate functional mutations in the *MYH9* gene, together with the absence of known neutral or selective evolutionary forces which could reasonably drive variants at this locus to high allele frequency. In addition, a high frequency of purported risk association variants in populations who do not share the high risk [26,27] pointed to the possibility that other neighboring genes might be responsible for the detected MALD signal. An answer to the riddle finally emerged from two cardinal works that identified missense mutations in the last exon of the *APOL1* gene, which happens to be the neighboring gene of *MYH9*: rs73885319 (S342G) and rs60910145 (I384M), a haplotype termed “G1” [28,29] (as opposed to the WT sequence “G0”). While, in the first study [28], strong signals were identified in additional neighboring genes, such as rs11089781 (Q58X) in the *APOL3* gene, the second study [29] pinpointed a strong second signal coming from another mutation in *APOL1*, rs71785313, an in-frame 6 base-pair deletion (N388del:Y389del) termed “G2”, which had also been noted in [28] but with low sample numbers. Notably, the G1 and G2 allelic variants (fittingly named “renal-risk alleles”) were not only more prevalent in African Americans in comparison to European Americans, but demonstrated an increased odds ratio for FSGS, HIV-1–associated FSGS, and end-stage kidney disease (ESKD) when two of them are inherited in comparison to one or zero risk alleles. The list of kidney diseases has been expanded and is now under the rubric of a new class designated APOL1 mediated kidney disease (AMKD) [30], and even extends to non-kidney phenotypes [31]. Moreover, the MYH9 kidney disease risk association signals were nullified upon conditioning for the *APOL1* risk alleles. The absence of a discernible difference in risk association between zero and one risk allele genotypes for G1 or G2, suggested a recessive pattern of kidney disease risk inheritance, raising a conundrum of inconsistency with a gain of function kidney injury risk mechanism in the African American study groups [29,32]. However, more recent reports examining the effect of G1 and G2 among continental West Africans pointed to both bi- and mono-allelic increased hazard ratio (HR) compared to zero risk alleles, albeit significantly lower than the HR observed in African Americans [33]. Furthermore, when tested for trypanolytic activity, serum from people harboring any of the three haplotypes (G0, G1, or G2) was sufficient to lyse *T. b. brucei;* however, only serum from those having at least one of the risk alleles was able to lyse *T. b. rhodesiense*, indicating that G1 and G2 are effectively gain-of-function acquired mutations [29]. This conclusion is further validated by the reported absence of a kidney health phenotype in an individual with a null state for APOL1 [34]. Also, since SRA was shown to lose its ability to bind the C-terminal domain of APOL1 only on a G2 background but not on G1, this suggested that these haplotypes might have differential functional capacities to maneuver around the resistant *T. b. rhodesiense* strain. Abstrusely, *T. b. gambiense* was resistant to all three haplotypes in vitro, but infection is contained and latent in individuals with even one G1 allele [29].

This tantalizing discovery that G1 and G2 represent gain-of-function alternatives to G0, endowing APOL1 with the ability to extirpate otherwise serum-resistant T. b. subspecies, set off a flurry of studies to examine the possibility that they might concomitantly be ‘toxic’ gain-of-function versions of G0, accompanying propensity to promote kidney injury, which was the basis for their discovery. While APOL1’s mode of action in trypanolysis has garnered a comfortable consensus, the way it causes host cellular toxicity has been confounded by reports of often contradictory experimental findings. Initially, owing to a high similarity between a peptide region within APOL1 sequence and BH3 domain, a short peptide motif found in certain BCL-2 family proteins, it was surmised that APOL1 constituted a novel Bcl-2 homology domain 3 (BH3)-only protein capable of triggering autophagic cell death [35]. However, protein–protein interaction studies demonstrated no physical interaction between APOL1 and any pro-survival proteins [36], and additional studies denoted that APOL1’s putative BH3 domain is dispensable for cellular toxicity [37]. Although some argued that a similar mode of action (i.e., lysosomal swelling and disruption) accounts also for cellular toxicity [38], others asserted that mitochondria are the prime target of APOL1 toxicity leading to mitochondrial dysfunction [39], fission [40], or increased permeability [41]. Vesicular [42] and endosomal [43] trafficking have been shown to be affected by APOL1 risk variants, but so has the endoplasmic reticulum [44,45,46]. Others took the ‘lysosomal pore-forming’ formulation to the next level and suggested that APOL1, the secreted protein, constitutes a bona fide ion channel in the plasma membrane of cells, promoting irregular ion currents that lead ultimately to osmotic cell swelling and death, albeit with different ions proposed as principal initiators of this cascade [37,47,48,49,50,51]. This discordance may be attributed, in certain cases, to the use of different cellular systems including ectopic overexpression or the reliance on artificial systems that might not adequately mimic the native context in which APOL1 operates. Whether many of these proposed causes of cellular injury might eventually collapse into a single (i.e., most proximate) trigger of cell death remains to be determined.

**Figure 1:**
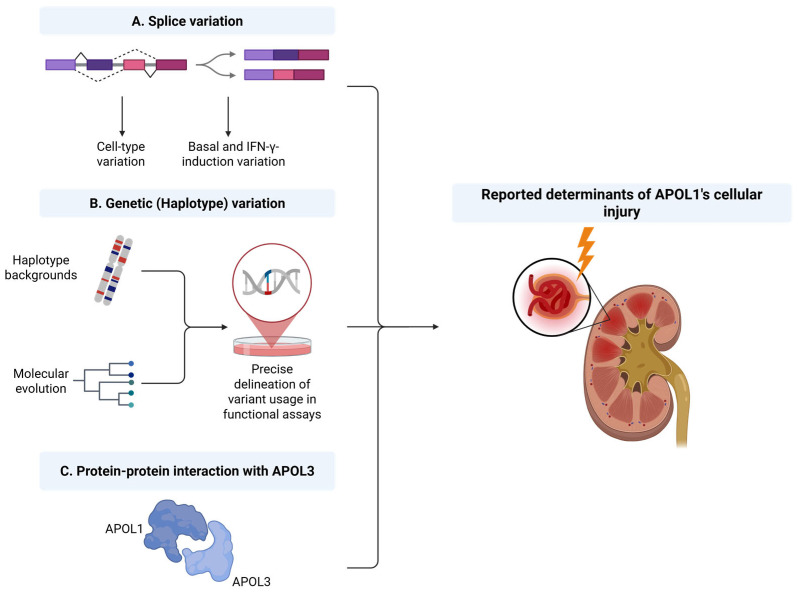
Primary aspects investigated in the study. An explicative figure depicting three domains previously reported to affect the expressivity of APOL1’s cytotoxicity: splicing, genetic variation, and protein–protein interaction with APOL3.

Host–pathogen interactions are the major driver of positive selection in humans [52], and as evident in *APOL1* evolution in respect to G1 and G2, mutations that conferred the best fitness and protection against invading trypanosomes allowed them to become increasingly common in the gene pool. Thus, deeper examination of *APOL1* genetic variants and their evolutionary trajectories should provide additional insights into their comparative modus operandi as trypanolytic factors, but also to their corresponding ‘collateral’ detrimental function in host cellular injury. Another confusing observation about AMKD is its incomplete penetrance and variable expressivity [53], suggesting one or more modifying influences and second ‘hits’ that might regulate APOL1 expression and/or function and thus the manifestation of the disease. Beyond cytokine mediated stimulation of expression, predominantly by IFN-γ, which has been linked to high expression levels of APOL1 and consequently to kidney injury [54,55], other modifiers, more basic and intrinsic to APOL1 biology, have been proposed to modulate its cytotoxicity. One such modifier is the haplotype background on which the renal-risk variants are present, further cementing the importance of thoroughly examining APOL1 genetic variation [56,57]. Another determinant was proposed to lie within *APOL1* splice variation, firmly coupling cytotoxicity to particular protein isoform products of the gene [58,59]. Protein–protein interactions with another member of the APOL gene family, APOL3, have also been proposed as an interaction potentially modified by the renal-risk variants G1 and G2, bringing about cellular damage [60,61]. The disparity in the different proposed mechanisms of cytotoxicity highlights a profound gap in the understanding of basic biology of APOL1 variant-driven cellular injury, warranting a deeper look into these basic mechanistic aspects that proposed to govern how cellular injury ensues. Accordingly, we performed cellular assays to assess the conditional expressivity of different *APOL1* splice variants, and conducted a comprehensive review of *APOL1* genetic variants, showcasing the apparent phylogenetic order of their evolution, coupled with bioinformatic analysis examining the basis for possible positive selection. Finally, in silico prediction tools and biological assays were used to reevaluate putative protein–protein interactions between APOL1 and APOL3, in relation to the renal-risk variants, G1 and G2 (Figure 1).

## 2. Materials and Methods

### 2.1. Cell Lines and Cell Culture

RCC 786-O (CRL-1932, ATCC, Manassas, VA, USA) and its isogenic derivatives were grown in RPMI medium (30-2001™, ATCC, Manassas, VA, USA), human immortalized podocytes in DMEM medium (30-2002™, ATCC, Manassas, VA, USA), HepG2 cells (HB-8065™, ATCC, Manassas, VA, USA) in EMEM medium (30-2003™, ATCC, Manassas, VA, USA), and HEK293-FT cells (Invitrogen, Waltham, MA, USA) in DMEM medium (# 10569010, Gibco, Waltham, MA, USA) all at 37 °C in 5% CO_2_. All media were supplemented with 10% fetal bovine serum (# F7524, Sigma, Saint Louis, MO, USA), and 1× penicillin–streptomycin (# P0781, Sigma, Saint Louis, MO, USA). The medium of HEK293-FT cells was additionally supplemented with 1× MEM Non-Essential Amino Acids Solution (# 11140050, Gibco, Waltham, MA, USA). Mycoplasma was routinely monitored by MycoStrip^®^—Mycoplasma Detection Kit (Invivogen, San Diego, CA, USA). Cells were passaged for up to ten passages before being discarded and replaced by freshly-thawed cells.

### 2.2. RNA Extraction and PCR Analysis

Cells were grown in 6-well culture plates (Lifegene, Mevo Horon, Israel) in duplicates until they reached ~70% confluency. The medium was then replaced by fresh pre-warmed medium. For stimulated cells, the medium was supplemented with 50 ng/mL human IFN-γ (# 130-096-873, Miltenyi Biotec., Bergisch Gladbach, Germany). The cells were cultured for additional 24 h. For siRNA-transfected cells, 18–24 h after transfections the cells were treated with 50 ng/mL IFN-γ for additional 1–3 days. Then, total RNA was extracted using a Quick-RNA Miniprep Kit (Car# R1054, ZYMO RESEARCH, Irvine, CA, USA) according to the manufacturer’s protocol and analyzed with Thermo Scientific™ (Waltham, MA, USA) NanoDrop™ for quantity and quality (A260/A280, A260/A230: >1.8). Using random primers, cDNA was synthesized using a ProtoScript^®^ II First Strand cDNA Synthesis Kit (Cat# E6560S, NEW ENGLANDS Biolabs, Ipswich, MA, USA). For PCR, Phusion^®^ High-Fidelity PCR Master Mix with GC Buffer (Cat# M0532L, NEW ENGLANDS Biolabs, Ipswich, MA, USA) was used to amplify APOL1 splice variant transcripts from 1 µL of the cDNA reaction according to the kit’s instructions. For siRNA-transfected cells, mRNA transcripts of *APOL3* isoform 1 were amplified from 10 ng of total RNA in a direct one-step RT-PCR reaction using a Transcriptor One-Step RT-PCR Kit (Roche, Basel, Switzerland). Samples were mixed with ExcelDye™ 6X DNA Loading Dye (SMOBIO Technology, Inc., Hsinchu City, Taiwan) 1:6 and loaded into a 2% Agarose (SeaKem^®^ LE Agarose, Lonza, Basel, Switzerland) gel. Images were obtained by the LAS-4000 Imaging System (Fuji, Tokyo, Japan), Table 1.

### 2.3. Plasmid Construction and Transfections

Synthetic DNA of isoform vA was produced and cloned into the pTwist CMV BetaGlobin WPRE Neo plasmid by Twist Bioscience. This plasmid was used as a template to create plasmids encoding isoforms vB and vC by means of PCR mutagenesis using Q5^®^ site-directed mutagenesis kit (E0554, NEW ENGLAND Biolabs, Ipswich, MA, USA) as per the kit’s protocol. The sequence of each construct was verified by Sanger sequencing. For transfection, HEK293-FT cells were seeded in duplicates/triplicates in a 6-well plate 48 h before transfection, so that they reach 80% confluency on the day of transfection (approx. 225,000 cells per well). Plasmid (1–1.5 µg per well) was used for transfection, using Lipo3K Transfection Reagent (APExBIO, Houston, TX, USA) according to manufacturer’s instructions. The medium was replaced with low-FBS containing serum ~5 h after transfection for continued overnight culture.

For siRNA transfections, 30 pmol of either a non-targeting siRNA probe (NT-siRNA) (Stealth RNAi™ siRNA Negative Control Med GC Duplex #2, Thermo Scientific™, Waltham, MA, USA) or an *APOL3*-targeting probe (siAPOL3) (apoL3 siRNA (h): sc-60193, Santa Cruz, Dallas, TX, USA) were reverse transfected into 300,000 HepG2 cells in one well of a 6-well plate using Lipofectamine RNAiMAX (Thermo Scientific™, Waltham, MA, USA) according to the manufacturer’s protocol.

### 2.4. Quantification of APOL1 Efflux by ELISA

Beginning 24 h after transfection initiation, supernatants were harvested then centrifuged at 1000× *g* for 4 min at 4 °C to pellet floating cells and cell debris. Then, 90% of the upper solution was passed through a 0.22 µm low-binding filter, aliquoted and stored at −80 °C until use. Supernatants were either used fresh, or thawed once from −80 °C storage, used and then discarded. Supernatants were diluted in ELISA Carbonate Coating Buffer (Thermo Scientific™, Waltham, MA, USA) 1:5. A total of 100 µL of each diluted sample was coated in triplicates on MaxiSorp high-binding ELISA plate (Thermo Scientific™, Waltham, MA, USA) overnight at 4 °C. The next day, wells were washed 3× with TBS + 0.05% Tween-20 (TBST), and blocked with TBST + 5% nonfat dry milk for 1 h at RT. After washing, 100 ul of primary anti-APOL1 antibody, rabbit oligoclonal 3.7D6/3.1C1 (a kind gift from Suzie J Scales, Genentech, San Francisco, CA, USA) diluted 1:12,000 in TBST was applied per well and incubated for 1 h at RT. After extensive washing, 100 μL of secondary HRP-conjugated anti-rabbit antibody (Jackson ImmunoResearch, West Grove, PA, USA) diluted 1:15,000 in TBST was applied per well and also incubated for 1 h at RT. After extensive washes, 50 μL of TMB substrate (1-Step™ TMB ELISA Substrate Solutions, Thermo Scientific™, Waltham, MA, USA) was added in each well, and incubated until good color development. The reaction was stopped by the addition of 50 μL Stop Reagent for TMB Substrate (Sigma, Saint Louis, MO, USA). The plate was read within 5 min with Biotek Gen5 plate reader (Agilent Technologies, Santa Clara, CA, USA) at 450 nm wavelength. Wells coated with supernatants of non-transfected cells served as a negative control for the assay.

### 2.5. Immunoblotting Analysis

Cells were grown in 6-well culture plates (Lifegene, Mevo Horon, Israel) in duplicates until they reached ~70% confluency. The medium was then replaced by fresh pre-warmed medium. For stimulated cells, the medium was supplemented with 50 ng/mL human IFN-γ (# 130-096-873, Miltenyi Biotec., Bergisch Gladbach, Germany). The cells were cultured for additional 24 h. Then, culture plates were removed from the incubator and placed immediately on ice, and cells were washed with ice-cold PBS. Cell extracts were prepared by incubating the cells for 30 min on ice with RIPA Lysis and Extraction Buffer (Cat# 89900, ThermoFisher) containing Halt™ Protease and Phosphatase Inhibitor Cocktail (Cat# 78440, Thermo Scientific™, Waltham, MA, USA). Cell extracts were cleared by centrifugation for 15 min at 15,000× *g* at 4 °C, and cleared samples were transferred to fresh tubes. Protein concentration was quantified using the BCA Protein Assay Kit (Cat# 71285, Merck Millipore, Darmstadt, Germany) according to the manufacturer’s instructions. For the analyzing the input lysate, equal amounts of protein per sample were taken into a new tube containing LDS Sample Buffer (Genscript, Piscataway, NJ, USA) and incubated at 70 °C for 10 min. For IP experiments, ~1 mg of cell lysate per sample was incubated with 10 μg of apoL3 Antibody (L07) (sc-101262, Santa Cruz, Dallas, TX, USA) in rotation overnight at 4 °C. The following day, 50 μL of pre-washed PureProteome™ magnetic protein G bead suspension (Merck Millipore, Darmstadt, Germany) was added per sample, and incubated in rotation for 40 min at RT. The beads were extensively washed 5 times with TBST, then incubated in LDS Sample Buffer at 70 °C for 10 min. Protein samples were separated on SurePAGE™ gels in Tris-MOPS-SDS Running Buffer (GenScript, Piscataway, NJ, USA), transferred onto polyvinylidene difluoride membrane (PVDF), and blotted with antibodies as indicated. Antibodies used were: rabbit oligoclonal 3.7D6/3.1C1 (a kind gift from Suzie J Scales, Genentech, San Francisco, CA, USA) 1:5000, mouse anti-α-Tubulin antibody (clone AA13, Sigma, Saint Louis, MO, USA) 1:2000, rabbit anti-APOL3 antibody (EPR8238(2), Abcam, Cambridge, UK) 1:1000. The incubation with primary antibodies diluted in filtered TBST + 5% BSA solution commenced overnight at 4 °C. After several thorough washes with TBST, membranes were incubated with the appropriate HRP-conjugated secondary antibody for 1 h at room temperature. Then, membranes were extensively washed with TBST, incubated for 40 s with Immobilon Crescendo Western HRP substrate (Cat# WBLUR0500, Merck Millipore, Darmstadt, Germany), and developed using the LAS-4000 Imaging System (Fuji, Tokyo, Japan). Upon capturing the chemiluminescent image, a colorimetric image was taken to denote the ladder. To preserve the quality of the chemiluminescent image of the immunoblot, the colorimetric image of the ladder was superimposed on it. The darker first lane in the immunoblot images of Figure 6 depicts the cropped, superimposed ladder. For full chemiluminescent and corresponding colorimetric images of the immunoblots, see Supplementary Figures S3–S5.

## 3. Results

### 3.1. Differential Splicing of Basal and IFNγ-Induced APOL1 Across Different Cell Types

It has been suggested that splicing processes provide a generalized explanation for the surprisingly small number of genes required to encode the complexity of living organisms [62]. The *APOL1* gene is encoded by seven exons that can be differentially spliced to give rise to multiple protein isoforms. While multiple splice variants have been proposed [13,63,64], only three verified protein isoforms can be found in the Universal Protein Knowledgebase (UniProt) [65]: Isoform O14791-1 or vA, O14791-2 or vB, and O14791-3 or vC. Baseline expression levels of *APOL1* transcripts can be found in many tissues [14]; however, a myriad of studies have shown that its expression can be exponentially induced in response to interferon gamma (IFN-γ) in numerous cell types [66,67,68,69,70]. Since several studies have suggested that APOL1 cell injury might be the quintessential result of one particular protein isoform [58,59], fully comprehending the landscape of *APOL1* splicing patterns, both basally and under induction, should provide not only valuable fundamental molecular biology insights, but also be a substantial advantageous step to developing therapeutics that can mitigate its cytotoxicity more precisely and efficiently [71].

**Figure 2:**
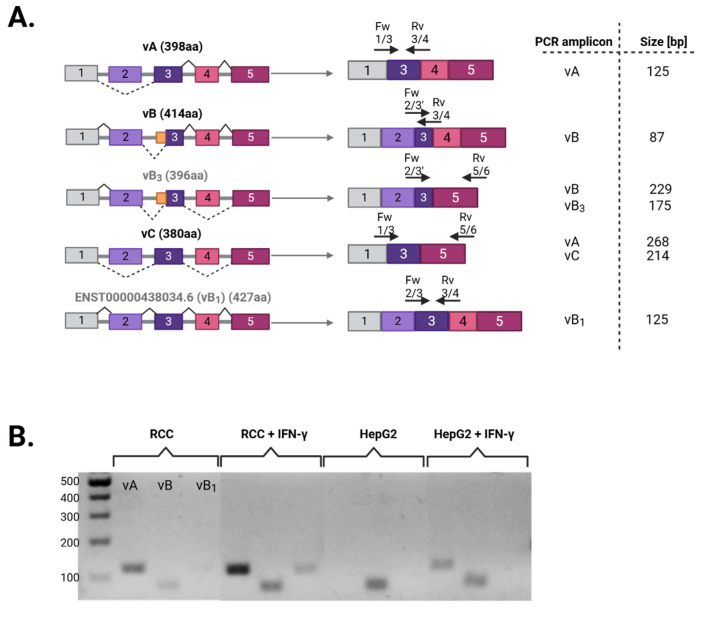

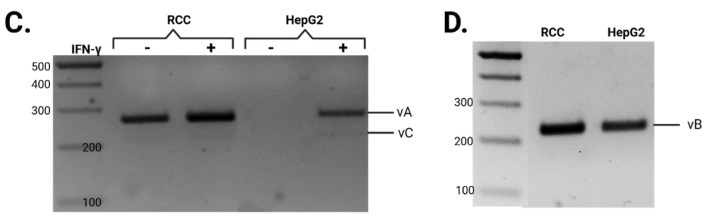
*APOL1* splice variation in different cellular systems and under basal or induced conditions. (**A**) Illustration depicting putative splice variants of *APOL1* (names shaded in gray are not reviewed/validated in UniProt), their final mature mRNA transcript, and the location of the exon–exon junction primers designed to delineate each splice variant. In the table to the right, the identities of the PCR amplicons and their predicted sizes are listed. Since exons 6–7 are present in all splice variants, they were omitted from the illustration. (**B**) Primer pairs that yield one definitive product were used to amplify their respective targets from total RNA extracted and reverse-transcribed into cDNA from RCC and HepG2 cells, with and without IFN-γ treatment. A representative agarose-gel image of the final PCR amplicons is presented, with markings above denoting the order of reactions in each segment: vA, vB, and then vB1. (**C**) Primer pairs that yield two amplicons of different sizes corresponding to vA and vC were used as in (**B**). A representative agarose-gel image of the final PCR amplicon depicts a very intense upper band (vA) and much fainter smaller band underneath (vC) following the same expression pattern. (**D**) A primer pair that yield two amplicons of different sizes corresponding to vB and vB3 was used as in (**B**,**C**). A representative agarose-gel image of the final PCR amplicon depicts only one intense upper band (vB) and no visible sign of vB3. Here both samples were treated with IFN-γ.

While there is no debate that exon 1 is not translated and that exons 5–7 are fully present in all protein isoforms, understanding of the splicing pattern of exons 2–4, and the different protein isoforms they consequently produce, remains more ambiguous. Considering that a signal peptide is encoded within these very exons, elucidating in which ways and under which conditions they are differentially spliced reflects not only on which protein isoform will be translated, but also probably on its localization, and hence, function. As illustrated in Figure 2A, isoform vA (O14791-1) is a product of fully splicing out exon 2 with a reading frame starting in the middle of exon 3, which is the same for isoform vC (O14791-3), except for the additional skipping of exon 4. In contrast, exon 2 is retained in isoform vB (O14791-2) with a reading frame starting almost at the end of it, and an alternative 3′ splice junction in exon 3, omitting many of its bases from the final transcript. Other splice variant iterations have been proposed for isoform vB, resembling it in retaining exon 2 and the reading frame start site, but either with concurrent skipping of exon 4, such as vB3 [63], or without the alternative acceptor site in exon 3, as in ENST00000438034.6 [72], also known as vB1. By utilizing different exon–exon junction-spanning primer pairs (Figure 2A), we investigated *APOL1* splice variation, both basally and upon IFN-γ induction, in two cellular systems: a human renal cell carcinoma (RCC) cell line, as a surrogate of the kidney, the primary site of APOL1’s cellular toxicity, and a hepatocellular carcinoma (HepG2) cell line, representative of the liver, the principal producer of serum APOL1 [73]. While mRNA transcripts of isoform vA were observed in RCC cells both basally and were elevated upon IFN-γ treatment, they were induced in HepG2 cells only after IFN-γ treatment. This was in contrast to isoform vB, the transcripts of which were present in both cell types at baseline, and surprisingly seemed to be induced by IFN-γ in kidney cells but not in hepatic cells. Moreover, variant ENST00000438034.6, or vB1, was RCC-specific, barely detectable at baseline but significantly induced by IFN-γ (Figure 2B). Since there are no primer pairs that would allow solely the specific amplification of isoform vC transcripts, we relied on amplicon size differences to distinguish between isoforms vA and vC. Like vA, trace amounts of a smaller-size mRNA transcript were observed, at extremely low levels in RCC cells, and only in response to IFN-γ in HepG2 cells (Figure 2C). We, like others [64], did not detect any vB3 transcripts in either cell type (Figure 2D).

**Figure 3:**
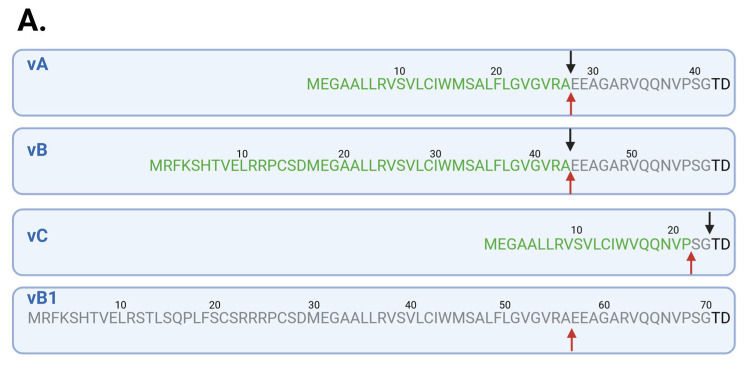

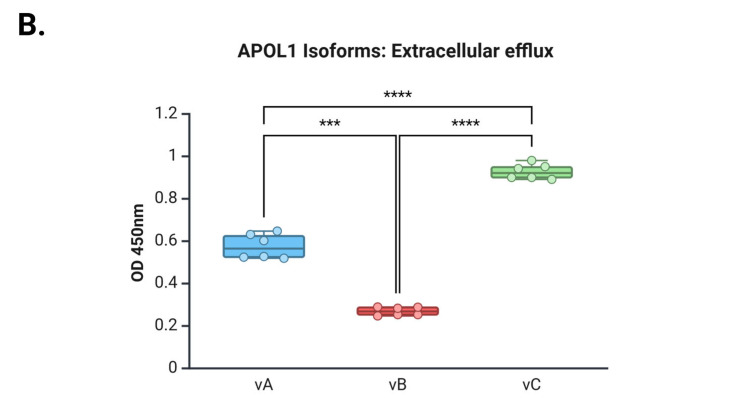
APOL1 isoform-dependent extracellular detection. (**A**) Illustration depicting the beginning of the amino acid sequence of all isoforms validated to be expressed as in Figure 2. In green is the predicted signal sequence of each isoform, only if predicted by both methods, with arrows indicating the predicted cleavage point (black arrow above by SignalP 6.0, and red arrow below by DeepTMHMM). In gray are ambiguous amino acids or those that are not shared by all isoforms, and in black are common amino acids. For isoform vB1 no signal peptide was predicted by SignalP 6.0, and only low-confidence SP was predicted by DeepTMHMM. (**B**) Representative ELISA results of measured APOL1 protein detection in the supernatant of HEK293 cells 24 h after transfection with a plasmid encoding the respective isoform of APOL1. Transfections were performed in duplicates, and each was coated as triplicates into ELISA plates. Welch’s one-way ANOVA with Dunnett T3 multiple comparisons test was used for statistical testing. W = 743.1400, DFn = 2.0000, DFd = 8.6120; *** and **** are both *p*-values < 0.0001.

After this clarification of differential splicing of exons 2–4, we further analyzed the putative secretory signal peptide each protein isoform might entail, which in turn would influence its localization and hence possibly also function. For in silico analysis of signal peptide and possible secretory capacity of each protein isoform, we utilized two protein language model-based algorithms, DeepTMHMM [74] and SignalP 6.0 [75]. Strikingly, apart from vB1, all APOL1’s isoforms were predicted to have the capacity to be localized to the ER luminal compartment and to be secreted, owing to a recognizable signal peptide (Figure 3A). To validate this experimentally, the coding sequence of isoforms vA, vB, and vC was cloned into a mammalian expression vector, transiently transfected into HEK293-FT cells, and the presence of APOL1 in the supernatant was examined 24 h later. In accordance with the in silico predictions, all three isoforms of APOL1 were detected in the supernatant, albeit isoforms vA and vC achieved higher levels of detection in the supernatant than vB (Figure 3B).

### 3.2. Evolutionary Chronology of APOL1 Haplotypes Reveals an Early Emergence of Toxic Gain-of-Function Variants and a Later Rise of Cell-Protective Ones

As already reported in the APOL-gene family discovery publication [13], two distinct background haplotypes of APOL1 have been differentiated based on variation of three single-nucleotide polymorphisms (SNPs), each found in complete linkage disequilibrium: Lys150/Ile228/Lys255 and Glu150/Met228/Arg255 (the numbering is according to isoform vA). Although the latter was less common, it was chosen as the ‘reference’ sequence of APOL1 in all major databases. Once APOL1, and its two risk alleles G1 and G2, came to attention, wider population-genetics initiatives [76] allowed the identification of additional SNPs in *APOL1* sequence, some with predicted and even experimentally proven deleterious or beneficial effects. Indeed, such wide population genetics efforts revealed that the 150/228/255 haplotype represents a prominent demarcation between African and European backgrounds. While Africans have a predominance of Glu150/Ile228/Lys255 (or ‘EIK’ in the single-letter code), Europeans preferentially have Lys150/Ile228/Lys255 (or ‘KIK’). The ‘reference’ sequence, Glu150/Met228/Arg255 (or ‘EMR’), while extremely uncommon in both populations, is more prevalent among Europeans. As with *APOL1* splice variation of protein isoforms, examination of phylogenetic branch delineating haplotype backgrounds has resolved several seeming inconsistencies in both population genetic and laboratory studies [77,78]. More specifically, the gain of cell injury effects of the G1 and G2 mutations are attenuated on the “reference” sequence background, in which they do not occur in natural populations. It remains to be determined whether the background haplotype effect is a direct consequence of one or more of the nucleotide differences or a reflection of the divergent phylogenetic history marked by the haplotypes. Another noteworthy revelation that came from large population genetics screens was the identification of protective SNPs, namely p.N264K, which when co-inherited with the high-risk variant G2, can significantly mitigate its cytotoxicity [79,80]. Nevertheless, also here, the underlying protective mechanism of this SNP has yet to be fully elucidated, partly as a result of the confounding influence in experimental studies of haplotype backgrounds, on which it never occurs in natural populations. Hence, a more thorough look into *APOL1* SNPs and on which haplotype backgrounds they occurred during evolution is not only warranted for performing correct and consistent biological functional assays, but is urgently needed to fully comprehend the chronological context in which toxic gain-of-function mutations arose in an ‘arms race’ against mutant versions of *T. brucei*, that possibly facilitated the subsequent emergence of counter-balancing protective ones—keeping in mind that the primary evolutionary pressure supporting spread of an allelic variant would be protection from pathogens affecting reproductive age groups rather than protection from kidney disease in mostly post-reproductive age groups. The balance of these forces and the phylogenetic order and time of emergence of these SNPs bears directly on their mechanism of kidney injury protection. Accordingly, we analyzed all *APOL1* SNPs in the 1000 Genomes Project phase 3, specifically in participants with African background, and particularly those occurring in exonic regions, and predicted to affect protein function. Specific attention was directed to SNPs of previously known haplotypes of interest, such as EMR/EIK/KIK, even those with extremely low mean allele frequencies. Despite the low frequencies, these vestiges of evolutionary history do advance insights. As presented in Table 2, we identified 18 different SNPs of interest, some of which are newly appreciated, and grouped them in reference to G0, G1, and G2 variants, with focus on the main haplotype in which they naturally, and in certain cases, exclusively appear (EMR/EIK/KIK). One such novel SNP was ‘EMK’, an intermediate haplotype between the reference ‘EMR’ and the African ‘EIK’, which was found in a few African participants. Another was the identification of the high-risk variant G1 on a ‘KIK’ haplotype in one donor, which was previously believed to occur exclusively on ‘EIK’ backgrounds. Moreover, we confirmed that the protective mutation, p.N264K, appears only with G0 or G2 and solely on the ‘KIK’ background (see Supplementary File S1 for full tables).

**Figure 4:**
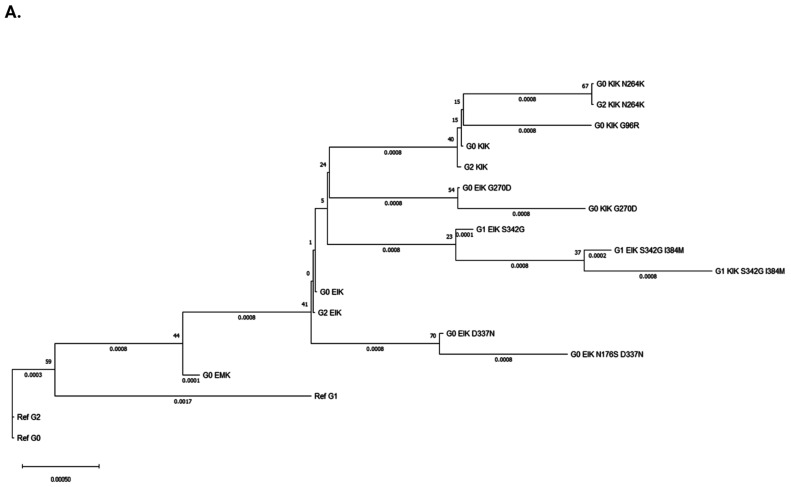

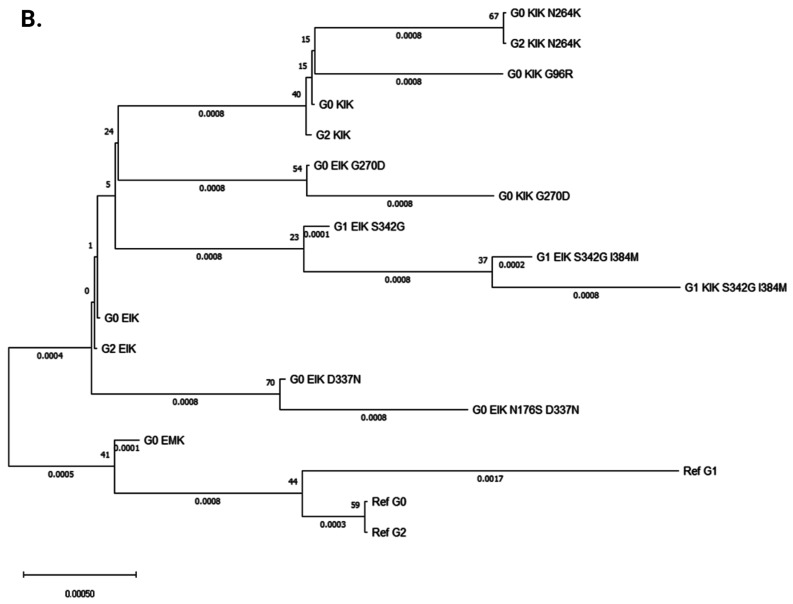
Phylogenetic analysis of *APOL1* variants and haplotypes. Phylogenetic tree constructed using all 18 sequences presented in Table 2 and inferred by the maximum likelihood (ML) method, with Kimura 2-parameter model used for substitution and Nearest Neighbor Interchange (NNI) as the ML heuristic method. Bootstrap values of 500 replications is depicted in front of each node. Analysis was conducted in MEGA11 [81], either by (**A**) fixing the reference G0 sequence as an outgroup, or (**B**) without a fixed outgroup.

Then, to map the network of *APOL1* haplotypes and to shed light on the evolution of these SNPs on their respective haplotype backgrounds through time, we conducted phylogenetic analysis. Given the small number of informative sites, several nodes were supported by relatively low bootstrap values. To compensate for this, we opted to use three different methods of phylogenetic analysis to ensure consistent topology of the phylogenetic tree (Figure 4 and Figure S1). All three methods confirmed that G0, G1 and G2 on the reference haplotype ‘EMR’ formed the basal clade of the dendrogram, whether G0 on the reference haplotype was fixed as the outgroup (Figure 4A and Figure S1A,C) or not (Figure 4B and Figure S1B,D). G0 on the ‘transitional’ haplotype ‘EMK’ was the first to bifurcate in a separate branch, and later, at the basis of another, yet larger cluster of branches, emerged G0, G1 and G2 on the ‘EIK’ background. Within this large lineage of branches, the topologies varied slightly between the three methods of analysis. However, consistently, G0 and G2 seem to cluster closer together, and to have emerged before G1, which branched separately away from them accumulating first p.S342G and only later the p.M384I mutation, and lastly appeared on the ‘KIK’ haplotype. In addition to the G1 sub-branch, three additional separate sub-branches appeared within this large lineage in close phylogenetic proximity: the emergence of G0 and G2 on the ‘KIK’ background, the p.D337N mutation with G0 on the ‘EIK’ background, and similarly the p.G270D mutation. Lastly, within the small G0/G2 ‘KIK’ sub-clade, emerged eventually the protective p.N264K mutation contemporary on both G0 and G2.

**Figure 5:**
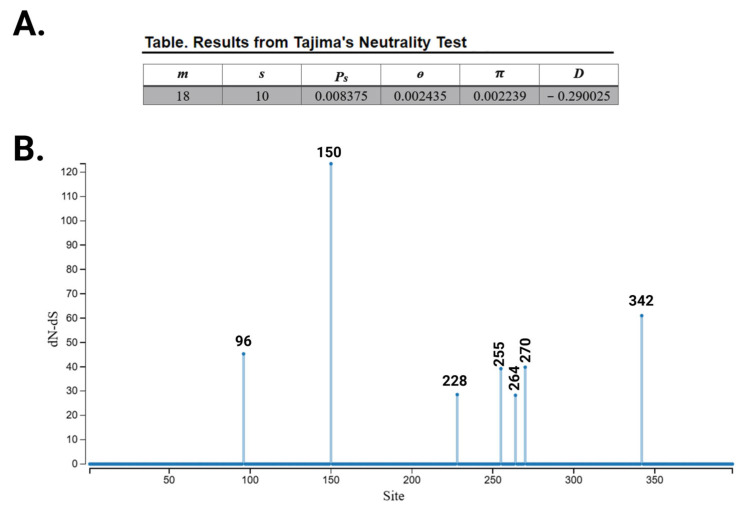

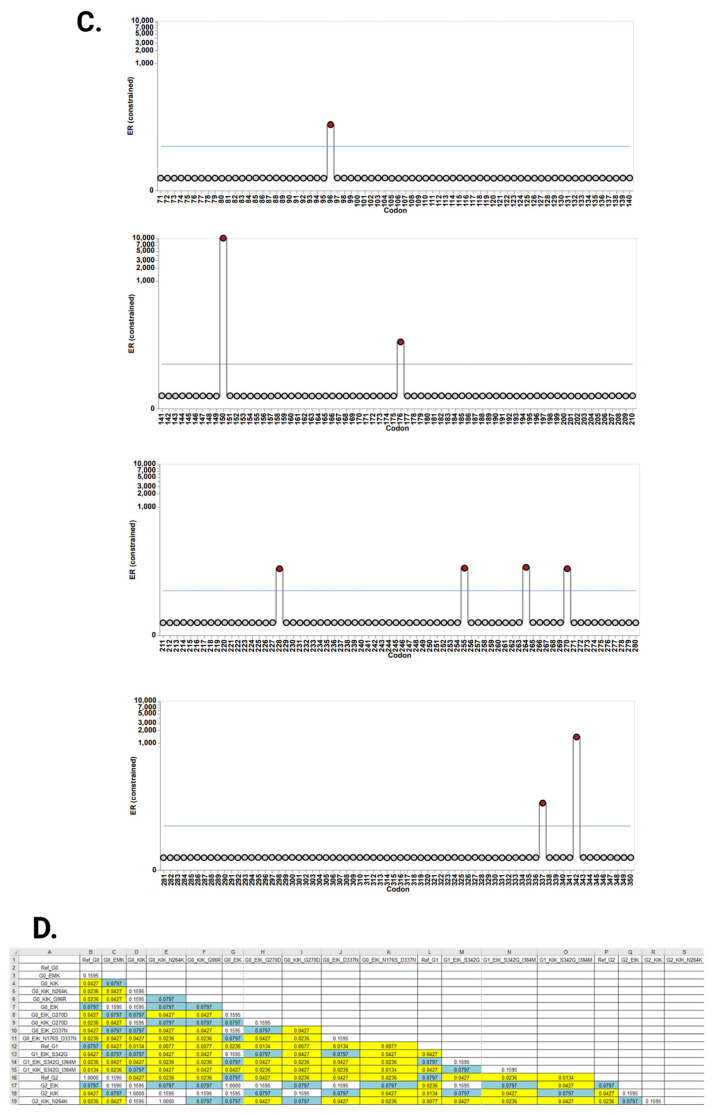
Positive selection analysis of *APOL1* variants. (**A**) Tajima’s neutrality test involving all 18 nucleotide sequences of *APOL1* variants presented in Table 2. Codon positions included were 1st+2nd+3rd. All ambiguous positions were removed for each sequence pair (pairwise deletion option). There were a total of 1194 positions in the final data set. Evolutionary analyses were conducted in MEGA11 [81]. *Abbreviations m* = number of sequences, *n* = total number of sites, *S* = Number of segregating sites, *P_s_* = S/n, *θ* = p_s_/a_1_, *π* = nucleotide diversity, and *D* is the Tajima test statistic. (**B**) SLAC analysis using a maximum likelihood ancestral state reconstruction and minimum path substitution counting. The test applied a simple binomial-based test of whether dS differs from dN. The estimates aggregate information over all branches, so the signal is derived from pervasive diversification or conservation. Amino acid positions of SNPs showing strong positive selection signal (i.e., high dN-dS) are denoted above each column. (**C**) BUSTED analysis using a random effects branch-site model fitted jointly to all or a subset of tree branches for alignment-wide evidence of episodic diversifying selection. Since an omega > 1 was observed, individual sites were subjected to evidence-ratio style analysis to explore which ones may have been subject to selection. ER ratio values are presented on the *y*-axis, and sites (amino acid position on the *x*-axis) above the threshold (blue line) are highlighted by red circles. (**D**) Codon-based test of positive selection for analysis between sequences. The probability of rejecting the null hypothesis of strict neutrality (dN = dS) in favor of the alternative hypothesis (dN > dS) (below diagonal) is shown. Values of *p* less than 0.05 are considered significant at the 5% level and are highlighted in yellow. The test statistic (dN-dS) is shown above the diagonal. dS and dN are the numbers of synonymous and nonsynonymous substitutions per site, respectively. The variance of the difference was computed using the analytical method. Analyses were conducted using the Nei–Gojobori method. This analysis involved all 18 nucleotide sequences of *APOL1* variants presented in Table 2. All ambiguous positions were removed for each sequence pair (pairwise deletion option). There were a total of 398 positions in the final dataset. Evolutionary analyses were conducted in MEGA11 [81].

Next, an assessment of whether any of these SNPs are (or were) a product of positive selection, like G1 and G2, was warranted. Given the lack of experimental data for most of these novel SNPs in regards to trypanosome killing, and to account for the possibility that such an assay would not be the appropriate method for potentially protective SNPs, like p.N264K [82], we relied on several in silico analysis methodologies to assess signatures of positive selection. While computing the extended haplotype homozygosity (EHH) is a preferred method to highlight aftermaths of positive selection on allele frequencies [83], EHH lacks statistical power when it comes to SNPs with low frequency, thus we relied on other tests which do not necessitate such a prerequisite. Tajima’s D test (D) is a classic neutrality test to detect natural selection in DNA sequences [84], and when applied to the DNA sequences of the 18 different variants of *APOL1*, a negative Tajima’s D of −0.29 was calculated, signifying excess of rare alleles possibly due to positive selection (Figure 5A). To verify which particular SNPs were a product of evolutionary pressure (on a per amino acid level) we relied on estimating site-specific synonymous (dS) and non-synonymous (dN) substitution rate parameters using different statistical tests. Single likelihood ancestor counting (SLAC) uses a maximum likelihood ancestral state reconstruction and minimum path substitution counting to estimate site-level dS and dN [85]. In addition to G1’s p.S342G that was used as a positive control, the test indicated that the genetic variation in six SNPs is caused by selective pressure, prime among them was p.E150K (Figure 5B). The Branch-site unrestricted statistical test of episodic diversification (BUSTED) test applies a random effects branch-site model fitted jointly to all or a subset of tree branches to test for alignment-wide evidence of episodic diversifying selection [86]. Although unlike SLAC, BUSTED provides gene-wide and not site-specific tests for positive selection, when there is evidence of positive selection, it can calculate “Evidence Ratios” (ERs) for each site, a descriptive parameter indicating whether a given site is subject to selection. ERs of eight SNPs were above the threshold (in addition to G1’s p.S342G) indicating positive selection of these variations (Figure 5C). Finally, a codon-based Z-test [87] was performed and confirmed that, in comparison to G0 on the ‘EMR’ reference background, for 12 out of the remaining 17 SNPs, the null hypothesis of strict neutrality (i.e., dN = dS) is rejected with great statistical significance, indicating positive selection of these SNPs (Figure 5D).

### 3.3. The Native Interaction Between APOL1 and APOL3 Is Modified by G1 and G2 Renal-Risk Variants

**Figure 6:**
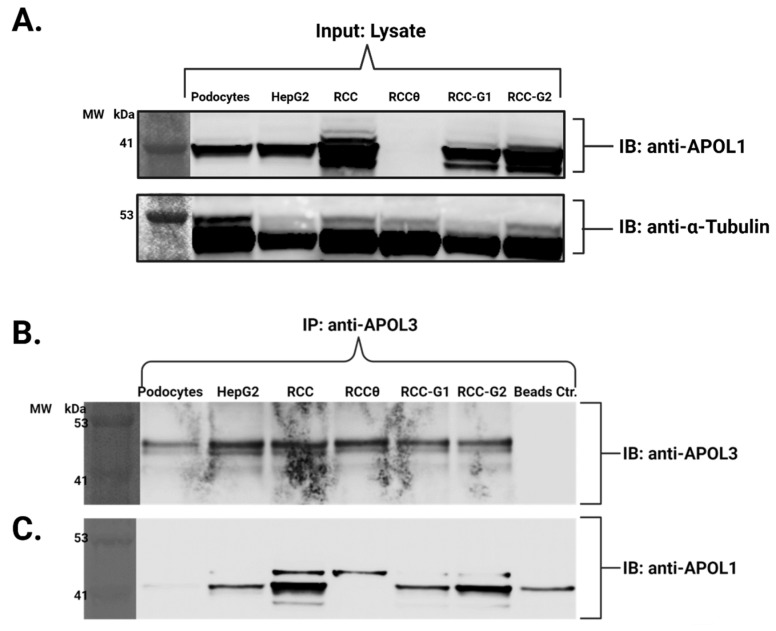
Differential APOL1-APOL3 native interaction revealed to be APOL1 haplotype- and cell type-dependent. Western blot analysis of cellular extracts of immortalized podocytes, HepG2, RCC and isogenic RCC cells expressing either G1, G2, or a null-variant of APOL1, after 24 h IFN-γ stimulation and subjected to immunoprecipitation by an anti-APOL3 antibody. (**A**) A fraction of the lysate was used to assess APOL1 expression levels in the input. Considering the expression of the normalizing protein α-Tubulin across samples, APOL1 was relatively expressed in the same manner across them. (**B**) The amount of APOL3 protein was evaluated in the eluates of all samples and in the beads-only control sample, to ensure equally effective IP procedure across samples. (**C**) The amount of APOL1 protein that was Co-IPed with APOL3 was assessed in the same eluates as in (**B**). A small thin band appears in the beads-only control sample indicating a slight ‘passive’ stickiness of APOL1 to the beads, albeit not in a way that would prevent solid biological interpretation of the results. Abbreviations: IP = immunoprecipitation, IB = immunoblotting, RCCθ = isogenic RCC cells with APOL1 null-variant, MW = molecular weight.

**Figure 7:**
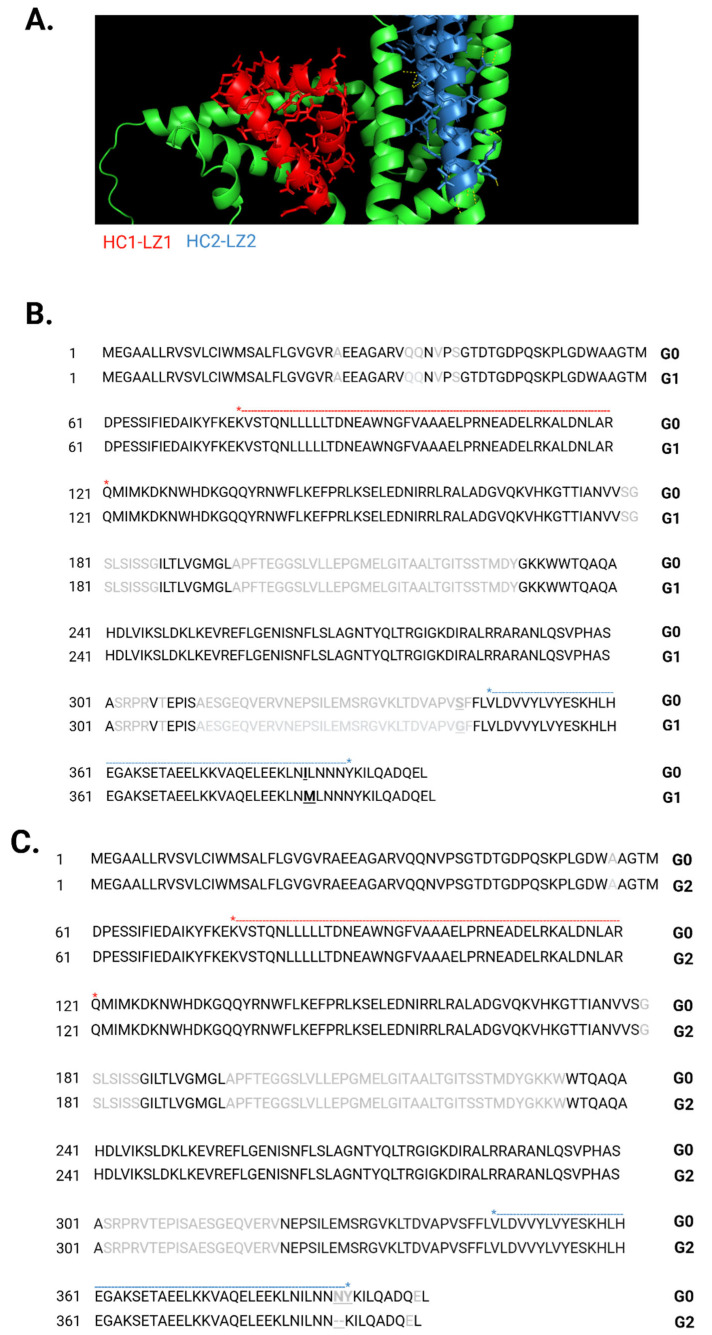
APOL1 G0, G1, and G2 protein folding predictions, alignment and inter-domain interactions. The structure of G0, G1, and G2 was predicted de novo using AlphaFold2. (**A**) Structure prediction of APOL1 G0 analyzed by PyMOL for all possible amino acid interactions within its chains. Depicted in red and blue are the proposed HC1–LZ1 and HC2–LZ2 domains, respectively. As it is evident from the illustration, they are predicted to be apart from each other, far beyond 4Å, the maximal distance that would allow amino acids to interact through their side chains. (**B**) Alignment of G0–G1 and (**C**) G0–G2 ensued afterward also using PyMOL. Amino acids in black are fully aligned in their predicted three-dimensional positioning, while shaded amino acids in gray are non-overlapping and spatially divergent between G0 and its two high-risk counterparts. In bold and underlined are the SNPs of G1 and G2, p.342 + p.384, and p.388–389, respectively. The upper red and blue lines demarcate the HC1–LZ1 and HC2–LZ2 domains, respectively, with the ‘*’ marking the first and last amino acid in the domain.

While APOL1 has attracted much scientific interest, given its fully articulated role in innate immunity against trypanosomes and its significant, yet still incompletely understood mechanistic association with kidney diseases, other members of the *APOL* gene family have yet to be equally explored and investigated. Prime among them is *APOL3*, which in one of the two renal-risk variant discovery papers [28], also showed a renal failure risk association of null variant of *APOL3* Q58X nonsense (rs11089781). This association was consolidated by two independent follow-up studies that showed significant association between a lack of functional APOL3 and increased risk for kidney disease [88,89], with only one of these [89] having the power to also demonstrate an APOL1 interaction effect. In addition, the relatively high sequence homology between APOL1 and APOL3, and the fact that both are transcriptional landing sites for IFN-γ regulatory factors and are considered interferon-stimulated genes (ISGs), further strengthen the theory that they might operate in coordination in host defense, and possibly intersect in the context of host cellular toxicity as a result of genetic variation, as suggested in the formulation of Pays and colleagues [90]. Indeed, recent investigations into APOL3’s role in innate immunity revealed that it acts, akin to APOL1, as a pore-forming lipid-binding protein, endowing host cells with a ‘detergent-like activity’ to restrict a menagerie of intracellular bacteria [91]. Unlike APOL1 however, it has high affinity to cationic lipids, and a strong aversion to cholesterol, such that its potent detergent activity is geared rather towards cationic lipid-rich, cholesterol-free bacterial membranes, away from the cholesterol-rich host cell membranes. More importantly, several studies have confirmed, albeit in non-native, recombinant fashion, a physical interaction between APOL1 and APOL3 with suggested regulatory ramifications on the function of both proteins, the disruption of which (by genetic variation), enables cellular toxicity to manifest [60,61]. To further explore a potential interaction between inherent, rather than ectopically overexpressed APOL1 and APOL3, and how it might be affected by the presence of the high-risk variants G1 and G2, we conducted co-immunoprecipitation (Co-IP) studies using several cellular systems, including an immortalized human podocyte cell line [92], HepG2 cells, RCC cells and isogenic RCC clones expressing APOL1-null, G1 or G2 variants [93]. First, to confirm the specificity of the antibodies used to IP or blot APOL3, we successfully conducted targeted knockdown of *APOL3* using siRNA, and managed to perfect and optimize the conditions to yield an almost complete knockdown of *APOL3* mRNA transcripts following IFN-γ induction (Supplementary Figure S2A). Then we used extracts of cells treated with either a non-targeting siRNA probe or the APOL3-siRNA probe, and conducted IP of APOL3 followed by APOL3 immunoblotting, showing that the band corresponding to isoform 1 of APOL3 was correspondingly missing from the siAPOL3 IP sample (Supplementary Figure S2B). Following this important confirmation, we performed the same regimen on the different cellular systems indicated above. First, we confirmed comparable expression levels of APOL1 protein among all cell lines in response to IFN-γ treatment (and lack thereof in APOL1-null RCC cells; Figure 6A). Then we validated the presence of comparable APOL3 protein levels in the eluates of all cell types following IP with anti-APOL3 antibody (Figure 6B). Finally, we verified the amount of APOL1 protein that was co-immunoprecipitated along with APOL3, and noted significant variation among the samples (Figure 6C). While the most prominent interaction between APOL1 and APOL3 was observed in RCC cells, this association was not detected in podocytes, and was only intermediate in liver cells. Of particular interest was the discovery of differential binding between both proteins across the different isogenic RCC clones, where WT APOL1 showed a strong interaction that declined with G2, and diminished even further with G1. In an effort to resolve the disjunction between this discovery and previous reports that also alluded to a differential binding between G0, G1 and G2 with APOL3 but in the opposite direction (G1 and G2 rather increase binding) [60,61], we focused on analyzing protein folding of APOL1 variants and of APOL3. A cornerstone of that proposal emanates from a surmised folding of APOL1 around two *cis*-interacting domains, each containing a tandem of a hydrophobic cluster of amino acids (HC) and a leucine-zipper domain (LZ), HC1-LZ1 and HC2-LZ2, which is disrupted by G1 and G2. To further scrutinize this possibility, we first utilized AlphaFold2 [94] to predict the three-dimensional structure of APOL1 variants (G0, G1, and G2) and of APOL3. Next, looking at the folding of APOL1 itself and using PyMOL (Molecular Graphics System, Version 2.0 Schrödinger, LLC.) to find all possible amino acid contacts within its domains, it became straightforward to deduce that the distance between the reported HC1-LZ1 and HC2-LZ2 domains is too far to allow any interactions between their amino acids side chains (Figure 7A). Then, we aligned the structures of G0, G1 and G2 using PyMOL to discern any differences in their folding. For the most part, the structures aligned very well, including HC1-LZ1 and HC2-LZ2. However, two domains did exhibit striking divergence in G1 and G2 from their counterparts in G0 (Figure 7B,C).

**Figure 8:**
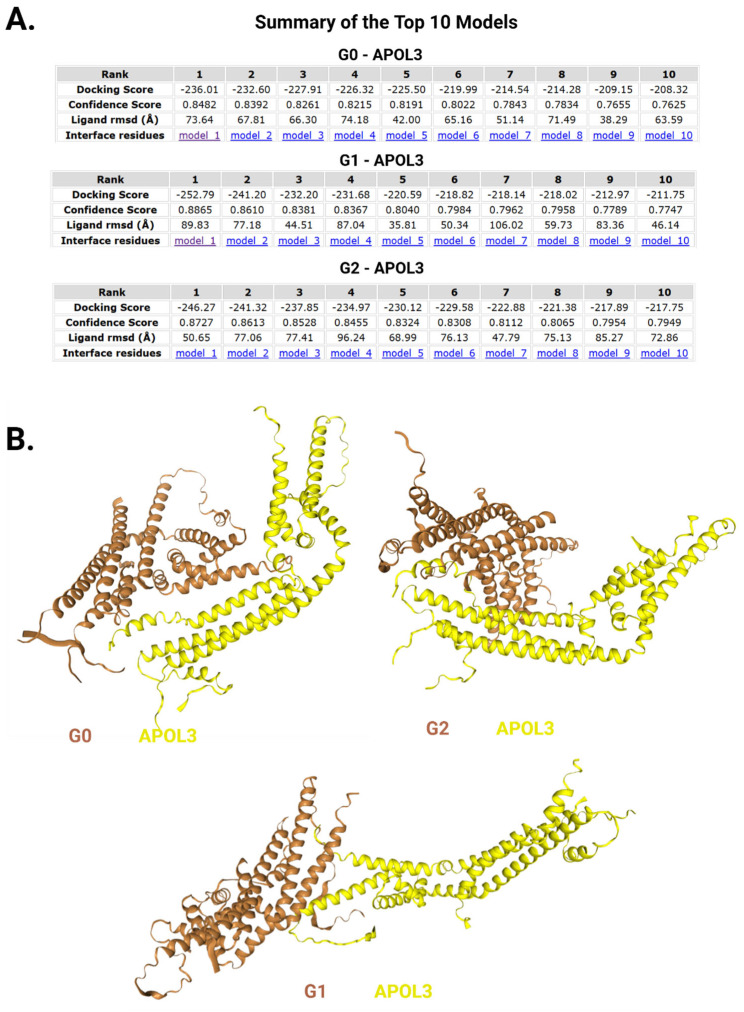
The interaction interface between APOL1 variants and APOL3 divulged by protein-protein docking computational analysis. AlphaFold2 predictions of APOL1 variants and APOL3 structures were uploaded to the HDOCK server for the evaluation of their interaction interface. (**A**) The top ten models of each interacting pair are presented in three separate tables, denoting the docking and confidence score of each model. (**B**) Evaluation of the top model of each pair revealed instantly visible significant changes of the interaction interface between both proteins, deviating markedly with G2, and almost entirely with G1. For precise list of interface residues see Supplementary File S2.

Finally, we employed the widely used HDOCK server (http://hdock.phys.hust.edu.cn/, accessed on 10 April 2025) [95] to perform protein–protein docking for APOL1 and APOL3 to pinpoint their surmised interaction interface, and assess whether it changes when G1 or G2 are at play. The software usually yields ten top predictions, and in all cases—G0-APOL3, G1-APOL3, and G2-APOL3—they were characterized by very good docking and high confidence scores (Figure 8A). We concentrated on the first top docking prediction for each pair and found a significant shift in the interaction interface, changing notably when G2 was modeled with APOL3 instead of G0, and even further, almost entirely different when G1 was modeled (Figure 8B). Correspondingly, 12 amino acids participating in the interface between G0 and APOL3 were retained by G2, and even strengthened by additional amino acids in their vicinity, while only 5 amino acids were savored by G1 (see Supplementary File S2 for full amino acids lists). Notably, none of the interfaces included any amino acids from the HC1-LZ1 or HC2-LZ2 domains of APOL1.

## 4. Discussion

In the space of a few thousand years—an instant in evolutionary chronology—a significant genetic variation has unfolded in the *APOL1* gene locus as a result of an evolutionary arms race between the host and its invading parasites (See Supplementary Table S1 for a full list of *Trypanosma strains* and their affiliated hosts and caused diseases). As the primary trypanolytic factor in human serum, two gain-of-function variants of APOL1, termed G1 and G2, have emerged as a consequence of the rise of serum-resistant trypanosome strains. This gain-of-function in trypanosome lysis, however, came with a concomitant liability: profoundly increased risk for kidney disease. While mechanism of trypanolysis has been elucidated, the latter, the unintentional collateral renal toxicity, is yet to be fully demystified and to achieve a consensus in terms of mechanisms and nuances [96]. This in no way detracts from the protection afforded by emerging therapies that inhibit APOL1 gain of injury currently in clinical trials [97]. It is often the case that therapeutic advance can precede comprehensive elucidation of molecular patho-mechanisms of the therapeutic target. Our review and results aim to identify and advance understanding of potential confounding complexities surrounding basic biological aspects of APOL1 that are reported to be detrimental to cellular viability, namely its splice variation, haplotype(s) backgrounds, and its interaction with APOL3.

One contributing factor to this ambiguity is splice variation of APOL1, as it has been proposed to be a major determinant of its cytotoxicity. This is even further highlighted since the discovery paper of APOL1 [12] regarded what is now known as isoform vB to be the main isoform of APOL1, and so did several earlier works investigating APOL1 cytotoxicity utilizing isoform vB for their studies [13,44,46]. Here, we investigated *APOL1*’s splice variation in two different cellular systems, renal and hepatic cells, and showed that isoform vB is found in both cell types basally, and is further induced by IFN-γ, while isoform vA was present in renal cells under both conditions, but only after stimulation in hepatic cells. We also confirmed the existence of isoform vC, which followed a similar expression pattern to isoform vA, revealed for the first time the exclusive expression of isoform vB1 in renal cells, and corroborated previous reports about the absence of the purported vB3 isoform. Although within the limitations of the cell lines used, the results still might indicate that isoform vB could be a major relevant isoform of APOL1 that is worthy of further study. Furthermore, we update the record about the efflux capacity of APOL1’s isoforms, which has been attributed exclusively to isoform vA, and provide both in silico and direct experimental evidence indicating that all three isoforms can indeed undergo efflux to the extracellular compartment. This discovery should revive interest in finding the true precursor identity of both APOL1 entities found in human serum [12,20], the first of which, based on N-terminal sequencing, might be the final product of both vA and vB isoforms after SP cleavage, while the second, the lower-molecular weight fragment, is closer to be a putative enzymatic output of isoform vC.

Another contributing factor to discrepancies around APOL1 cytotoxicity is the inconsistent use of haplotype backgrounds. The variation in this regard oscillated broadly, from some reporting that APOL1’s toxicity is commensurate to expression level of the protein, irrespective of haplotype background or genetic variant (risk vs. non-risk) [98], to others showing haplotype-dependent cytotoxicity [56,57], all the way to a few demonstrating cytotoxicity of the high-risk variants on all haplotype backgrounds [44,63,76]. Since some of the experimental systems used genotypes that do not occur in natural populations, we examined one of the largest human population genetics datasets, the 1000 Genomes Project phase 3, and identified, validated, and summarized all function-altering genetic variants of APOL1 coupled only with the haplotype backgrounds on which they naturally appear. In addition, through rigorous statistical testing, positively-selected variants were identified, and evolutionary inference of their chronology was provided. This analysis showed that p.E150K was the outcome of a possibly even stronger selective force than G1, and the haplotype it created (‘KIK’) emerged last on various pre-existing variants on the ‘EIK’ haplotype (G0, G2, G1, p.G270D). This is of particular interest to better understand the evolutionary path that projected the G2 protective p.N264K SNP, which has been recently proposed to be a product of a proximity recombination event between an ‘EIK’-G2 variant and a ‘KIK’-N264K counterpart [99]. The evolutionary trajectories shown here, in addition to the fact that other SNPs upstream (like p.G270D) and downstream (like G1) of the N264 position emerged on the ‘KIK’ background as well, do not support this proposition. Importantly, it will be necessary to understand the counterbalancing protection from kidney disease possibly conferred by the N264K event, which does not diminish *T. b. rhodesiense* trypanolysis in in vitro assays [82]. In African regions with a predominance of *T. b. gambiense*, whose clinical severity is worsened by G2 [100], the emergence of N264K might make evolutionary sense. In any case, the very strong inhibition of APOL1-G2 host cell injury conferred by N264K provides a possible avenue to mechanistic insight worthy of further investigation.

Finally, we revisited another potential course of complexity, namely intra-gene *APOL* family interactions, specifically between APOL1 and APOL3, that have been proposed to cast ‘regulatory’ shadows on APOL3’s and APOL1’s function, and by extension possibly cytotoxicity as well. The rise to high frequency of a null variant of APOL3 in African ancestry populations (despite the association of that variant with kidney disease), suggests a context specific adaptive advantage in what is otherwise considered a protective gene product. The more enigmatic APOL3 has been ascribed with innate immunity and pore-forming, lipid-binding proprieties, not only in restricting intracellular bacterial infections, but also in promoting phagosomal rupture and allowing cross-presentation of engulfed antigens onto MHC class I molecules in antigen-presenting cells [101]. Thus, it was a puzzling revelation to show that this innate immunity factor acts also as a docking platform for a multi-protein complex responsible for Golgi and actomyosin maintenance, the integrity of which is jeopardized by APOL1 high risk variants or a null variant of APOL3 itself [60,61]. This is not surprising, given the ability of complex biological systems to maintain old functions of genes and attain new ones after acquiring random mutations, and knowing that an ancient retrovirus insert may have contributed a new transcriptional start site for *APOL3*, allowing the expression of a new protein isoform [102]. We showed here, for the first time, direct experimental evidence of the native interaction between APOL1 and APOL3, which appeared to be both cell type- and haplotype-dependent. In renal carcinoma cells, this interaction was the strongest, while it was moderate in hepatic cells, and almost non-existent in human immortalized podocytes. If indeed APOL3 governs in some aspect APOL1’s function, this might explain why podocytes are more susceptible to APOL1 cytotoxicity. Moreover, in respect to APOL1 variants, G2 was shown to be a weaker binder than G0 to APOL3, and G1 was shown to be the weakest. In silico protein–protein docking analysis verified that the interaction interface between both proteins varies drastically across APOL1 variants G0, G1, and G2. However, G2 did retain more amino acids of the original G0-APOL3 in its interface with APOL3, and significantly more than G1. This corroborated well our former experimental observations, and refuted previous projections of the interaction interface between both proteins (i.e., through HC-LZ domains). Another explanation for this apparent disparity is utilizing here the native physiological interaction between APOL1 and APOL3 following concordant induction by IFN-γ, rather than using recombinant proteins and artificial systems that might not represent the true natural folding of the proteins.

The quest to provide refined preventive and therapeutic solutions for AMKD is enhanced by continued clarification of fundamental mechanisms underlying APOL1 host-cell injury. Our findings herein underscore the importance of taking into consideration several intrinsic biological aspects of APOL1 for better clinical evaluation of AMKD. For instance, since heightened inflammation with IFN-γ stimulation, which is linked to higher incidence of AMKD in predisposed individuals, is expected to increase APOL1 and APOL3 levels pari passu, this needs to be considered in interpreting the differential risk genotypes and also variable expressivity, as well as modifying effects of APOL3 stop/gain mutations. Furthermore, while several PCR- and NGS-based approaches to identify renal-risk variants G1 and G2 have been proposed to enter standard clinical care [103], our findings and others, suggest that full-gene sequencing may be needed to delineate the haplotype background on which these variants are present and thereby improve genomically precise patient management.

## 5. Conclusions

The current manuscript serves both to review the existing scientific literature and also to provide new research findings that continue to advance our understanding of APOL1 cytotoxicity. Much progress has been achieved in unravelling molecular mechanisms, interactions and pathways involved in APOL1 causation of gain of cell injury, and in turn its most prominent ostensible clinical manifestation—kidney disease risk. Nevertheless, “loose ends” remain, whose resolution will benefit patient management. We identify three such incompletely resolved domains, present new findings in relation to each, and point out the insights that emerge of relevance to advancing the current state of knowledge, with the hope that these will demystify some ongoing ambiguities and scientific debate in the near future.

## 6. Limitations

A major and under-emphasized theme of our experimentation was to maximize the utilization of experimental platforms with native and endogenous APOL1 and its genotypic variants and avoid ectopic overexpression or pure recombinant protein–protein interaction systems, which have yielded conflicting results in past studies. However, we do recognize that many of the results are based on work performed with renal carcinoma cells and HepG2 cells, which are cancerous transformed cell lines that might deviate biologically from healthy normal cells in respect to APOL1 biology. The need for studies in human podocyte systems with endogenous inducible APOL1 and APOL3 expression is thus vital and more appropriate. We do provide one such set of results in human podocytes and these do set the stage for future studies with podocytes differentiated from genome edited induced pluripotent stem cells or other sources with the genotype encountered in natural human populations. We also point out the technical limitation in immunoblot separation and distinction of splice isoforms.

## Figures and Tables

**Table 1:** Primers used for PCR are listed below.

# Primer	Sequence
*APOL1* Fw 1/3	GAAGATTCCTTGGAGGAGCACAC
*APOL1* Fw 2/3′	CACTGTGGAATTGAGGAGCACAC
*APOL1* Fw 2/3	CACTGTGGAATTGAGGAGGCCC
*APOL1* Rv 3/4	GTGCACTCATCCAGATGCAGAG
*APOL1* Rv 5/6	GATACTGCTCTCTGGGTCCATGG
*APOL3* Fw 1/3	GAGAAGCTTCCTTGAAAAGAAACGCTTTAC
*APOL3* Rv 4	CAAAACAAGACCAGCAAGGGAC

**Table 2:** *APOL1* variants and background haplotypes. All 18 sequences were retrieved from the 1000 Genomes Project phase 3, and were validated to be present in at least one donor in the data set. Ten variants appeared with G0 on different haplotype backgrounds, four with G1 and G2 each. Highlighted in bold red are amino acids that differ from the reference G0 ‘EMR’ sequence.

	Amino Acid Position	96	150	176	228	255	264	270	337	342	384	388–389
**1**	**Ref G0**	G	E	N	M	**R**	N	G	E	S	I	IN
**2**	**G0 ‘EMK’**	G	E	N	M	**K**	N	G	E	S	I	IN
**3**	**G0 ‘KIK’**	G	**K**	N	**I**	**K**	N	G	E	S	I	IN
**4**	**G0 ‘KIK’ N264K**	G	**K**	N	**I**	**K**	**K**	G	E	S	I	IN
**5**	**G0 ‘KIK’ G96R**	**R**	**K**	N	**I**	**K**	N	G	E	S	I	IN
**6**	**G0 ‘EIK’**	G	E	N	**I**	**K**	N	G	E	S	I	IN
**7**	**G0 ‘EIK’ G270D**	G	E	N	**I**	**K**	N	**D**	E	S	I	IN
**8**	**G0 ‘KIK’ G270D**	G	**K**	N	**I**	**K**	N	**D**	E	S	I	IN
**9**	**G0 ‘EIK’ D337N**	G	E	N	**I**	**K**	N	G	**N**	S	I	IN
**10**	**G0 ‘EIK’ N176S D337N**	G	E	**S**	**I**	**K**	N	G	**N**	S	I	IN
**11**	**Ref G1**	G	E	N	M	R	N	G	E	**G**	**M**	IN
**12**	**G1 ‘EIK’ S342G**	G	E	N	**I**	**K**	N	G	E	**G**	I	IN
**13**	**G1 ‘EIK’ S342G I384M**	G	E	N	**I**	**K**	N	G	E	**G**	**M**	IN
**14**	**G1 ‘KIK’ S342G I384M**	G	**K**	N	**I**	**K**	N	G	E	**G**	**M**	IN
**15**	**Ref G2**	G	E	N	M	R	N	G	E	S	I	***Del***
**16**	**G2 ‘EIK’**	G	E	N	**I**	**K**	N	G	E	S	I	***Del***
**17**	**G2 ‘KIK’ N264K**	G	**K**	N	**I**	**K**	**K**	G	E	S	I	***Del***
**18**	**G2 ‘KIK’**	G	**K**	N	**I**	**K**	N	G	E	S	I	***Del***

## Data Availability

The original contributions presented in this study are included in the article/Supplementary Material. Further inquiries can be directed to the corresponding author.

## Supplementary Materials

The following supporting information can be downloaded at: https://www.mdpi.com/article/10.3390/cells14131011/s1, Figure S1: Additional phylogenetic analysis of APOL1 variants; Figure S2: Confirmation of anti-APOL3 antibodies specificity; Figure S3: Full immunoblot images and colorimetric image of Figure 6A in the main text; Figure S4: Full immunoblot images and colorimetric image of Figure 6B in the main text; Figure S5: Full immunoblot images and colorimetric image of Figure 6C in the main text; Table S1: Major trypanosoma species and their impact; Supplementary File S1: Full participant list of APOL1 SNPs in the 1000 Genomes Project; Supplementary File S2: Full amino acid list of APOL1–APOL3 interaction interface.

## Abbreviations

The following abbreviations are used in this manuscript:

AMKD	APOL1 mediated kidney disease
ApoAI	Apolipoprotein AI
APOL1	Apolipoprotein L1 (APOL1)
BH3	Bcl-2 homology domain 3
BUSTED	Branch-site unrestricted statistical test of episodic diversification
CKD	Chronic kidney disease
EHH	Extended haplotype homozygosity
ESKD	End-stage kidney disease
FSGS	Focal segmental glomerulosclerosis
HDL	High density lipoprotein
Hp	Haptoglobin
Hpr	Haptoglobin-related protein
HR	Hazard ratio
IFN-γ	Interferon gamma
ISG	Interferon-stimulated genes
MALD	Mapping by admixture linkage disequilibrium
SLAC	Single likelihood ancestor counting
SNP	Single-nucleotide polymorphism
SRA	Serum-resistance associated protein
TLF	Trypanosome lytic factor
VSG	Variant surface glycoprotein
