# Supplementary material for: Apolipoprotein L1 (APOL1): Consideration of Molecular Evolution, Interaction with APOL3, and Impact of Splice Isoforms Advances Understanding of Cellular and Molecular Mechanisms of Cell Injury
Source: Cells. 2025 Jul 2;14(13):1011. doi: 10.3390/cells14131011 (PMC12248677; doi:10.3390/cells14131011)
Supplement: Supplementary file 1 [file cells-14-01011-s001.zip › Supp Figures and Table S1.pdf]

Supplementary Figure S1

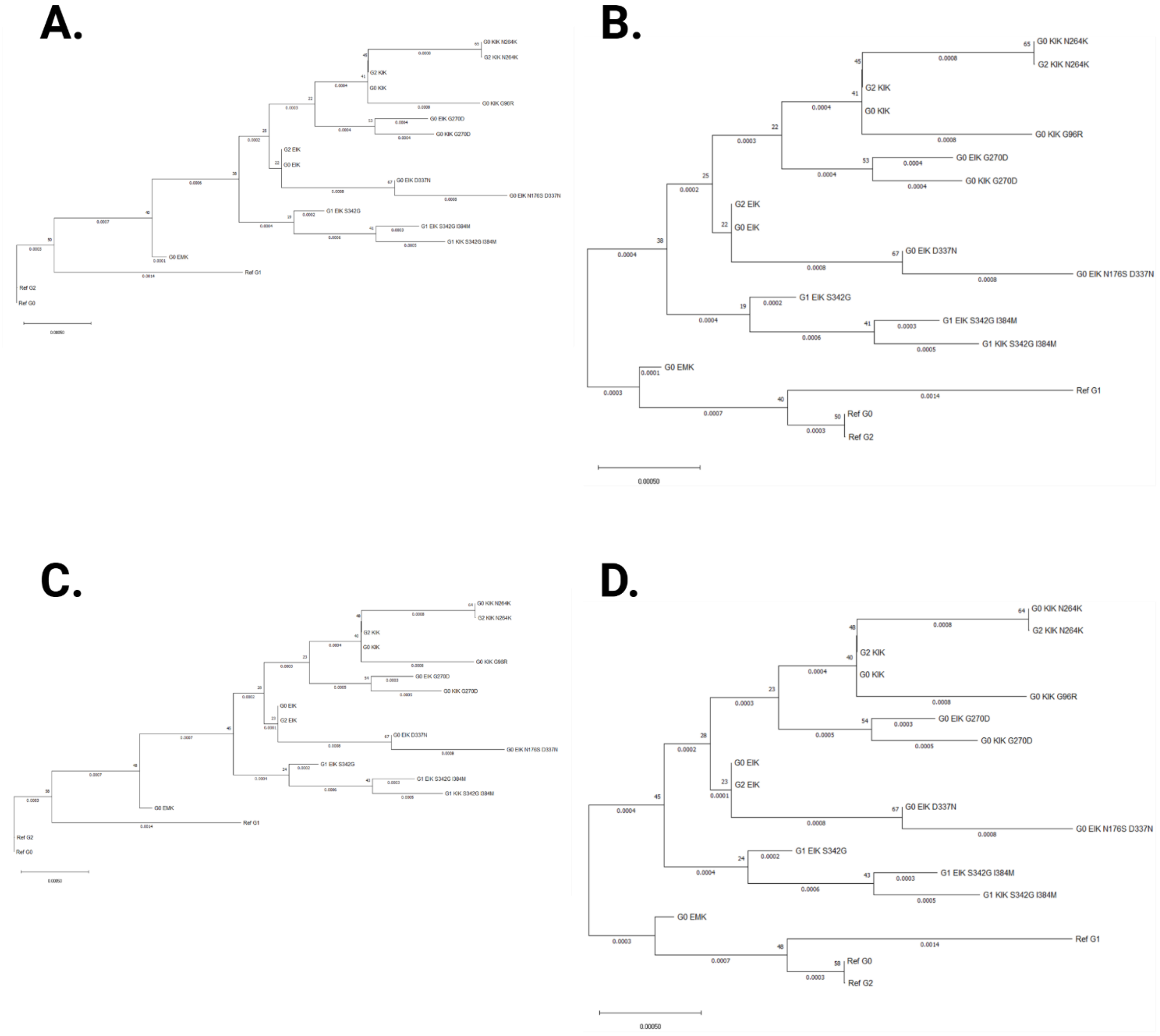

Supplementary Figure S1. Additional phylogenetic analysis of *APOL1* variants. Phylogenetic tree constructed using all 18 sequences presented in table 1 and inferred by the Neighbor Joining (NJ) method with Maximum Composite Likelihood used as a substitution model. Bootstrap values of 500 replication is depicted in front of each node. Analysis was conducted in MEGA11, either by **(A)** fixing the reference G0 sequence as an outgroup, or **(B)** without a fixed outgroup. Phylogenetic tree constructed using all 18 sequences presented in table 1 and inferred by Minimum Evolution method with Maximum Composite Likelihood used as a substitution model. Bootstrap values of 500

replication is depicted in front of each node. Analysis was conducted in MEGA11, either by **(C)** fixing the reference G0 sequence as an outgroup, or **(D)** without a fixed outgroup.

Supplementary Figure S2

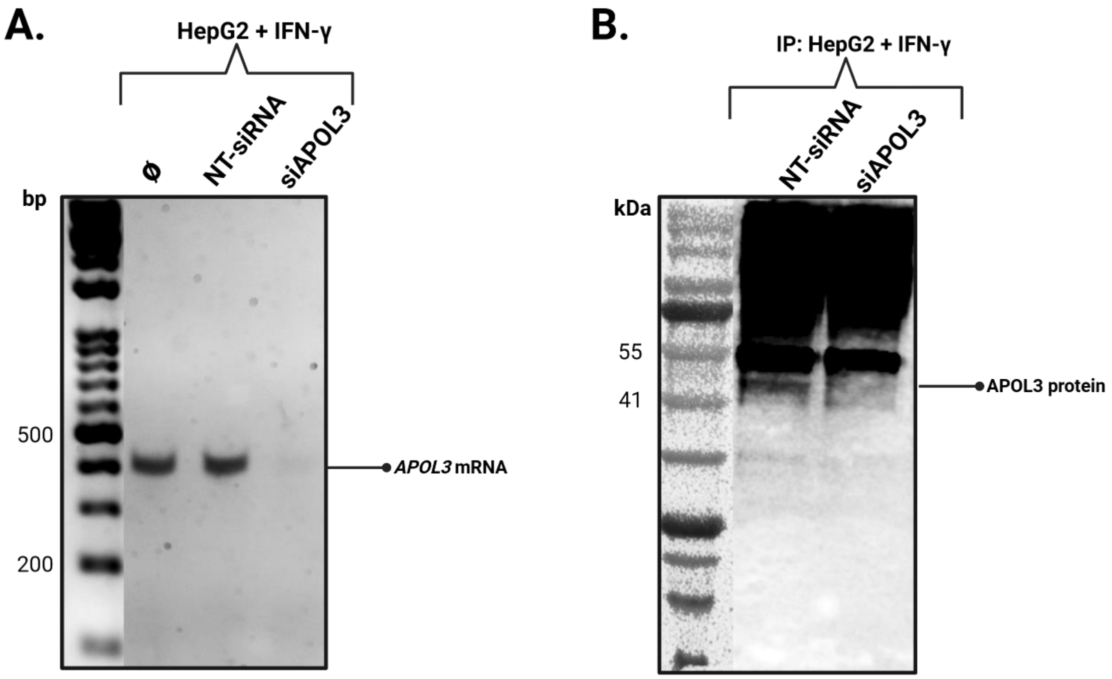

Supplementary Figure S2. Confirmation of anti-APOL3 antibodies specificity. To confirm the specificity of the antibodies used to IP and blot APOL3, targeted knockdown of *APOL3* was performed in HepG2 cells after IFN-γ stimulation using siRNA. **(A)** *APOL3* direct RT-PCR amplification from total RNA extracted from cells after 2 days of IFN-γ stimulation and 3 days of siRNA transfection: **ø** - non-transfected, **NT-siRNA** – transfected with a non-targeting siRNA, or **siAPOL3** – transfected with *APOL3*-targeting siRNA **(B)** The same IP regimen followed by the same immunoblotting step as indicated in Figure 6B in the main text was performed on cell lysates of 3 days IFN-γ-treated HepG2 cells following transfection with NT-siRNA or siAPOL3.

Supplementary Figure S3

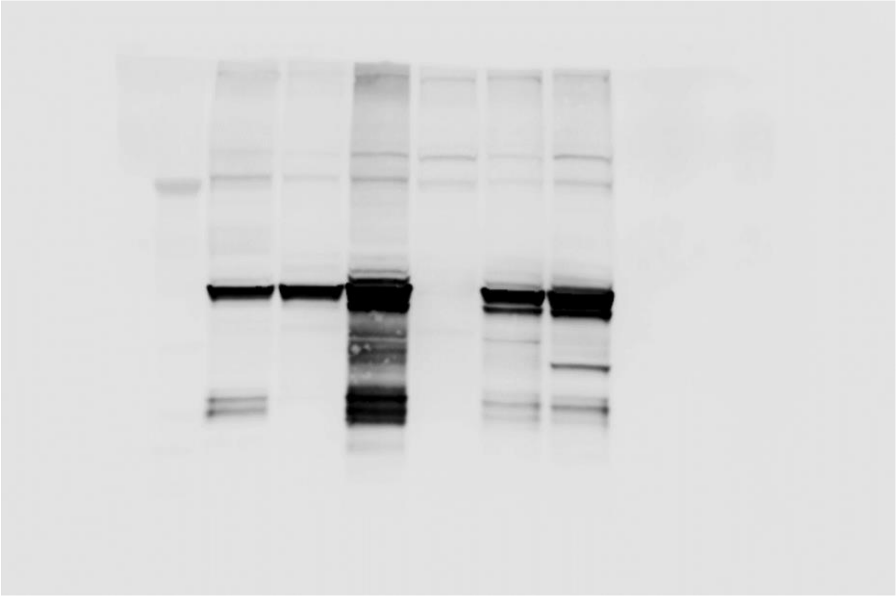

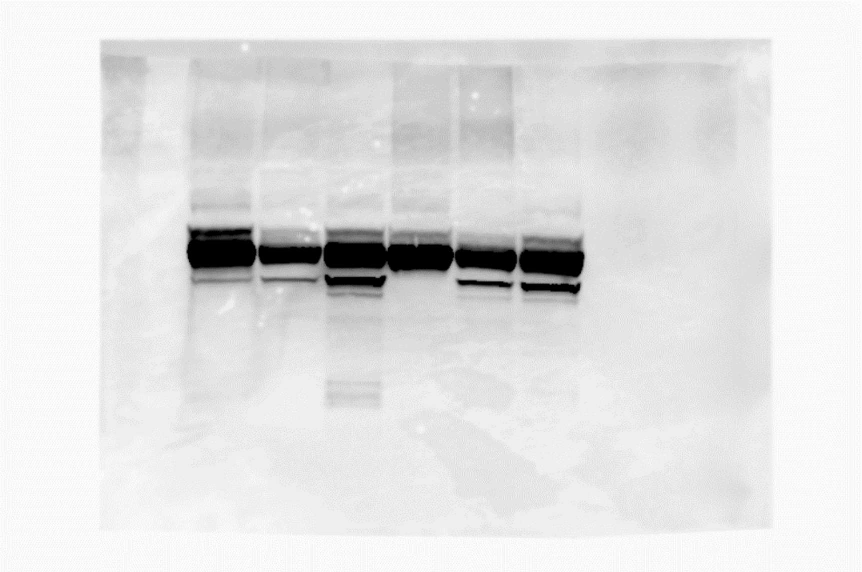

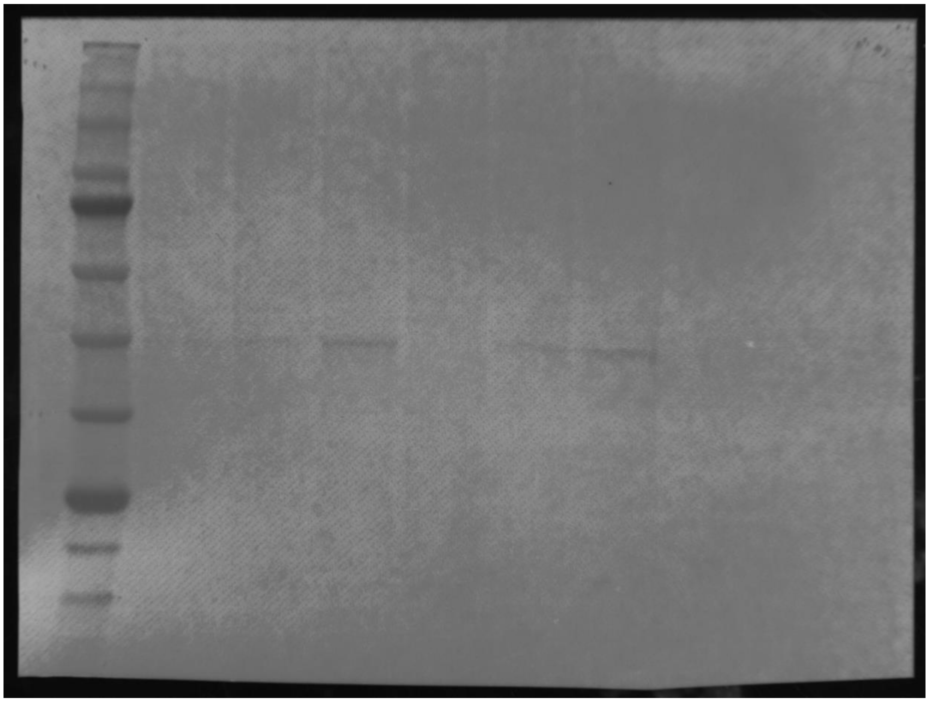

Supplementary Figure S3. Full immunoblot images and colorimetric image of Figure 6.A in the main text. Upper image = Blotted with Anti-APOL1; Middle image = Blotted with anti-α-Tubulin; Lower image = Colorimetric image indicating the ladder.

Supplementary Figure S4

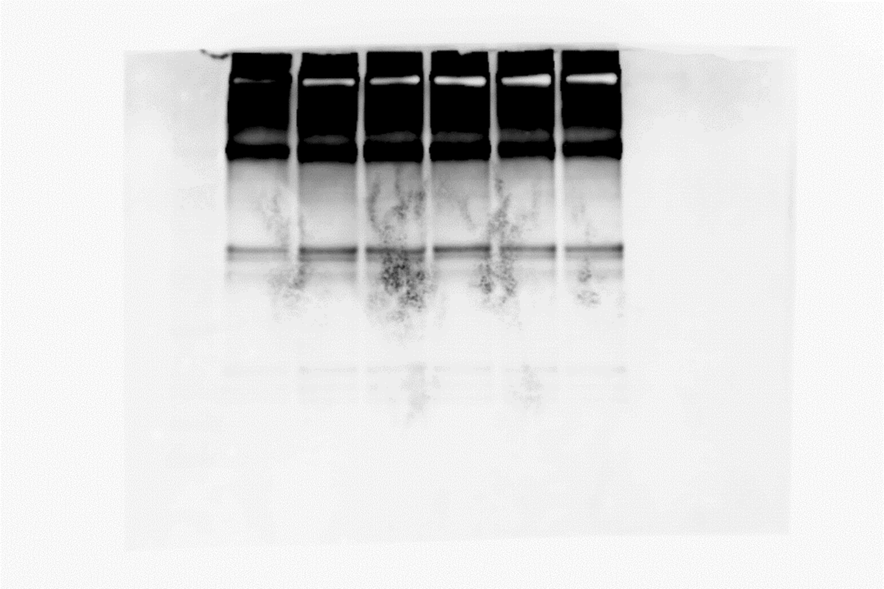

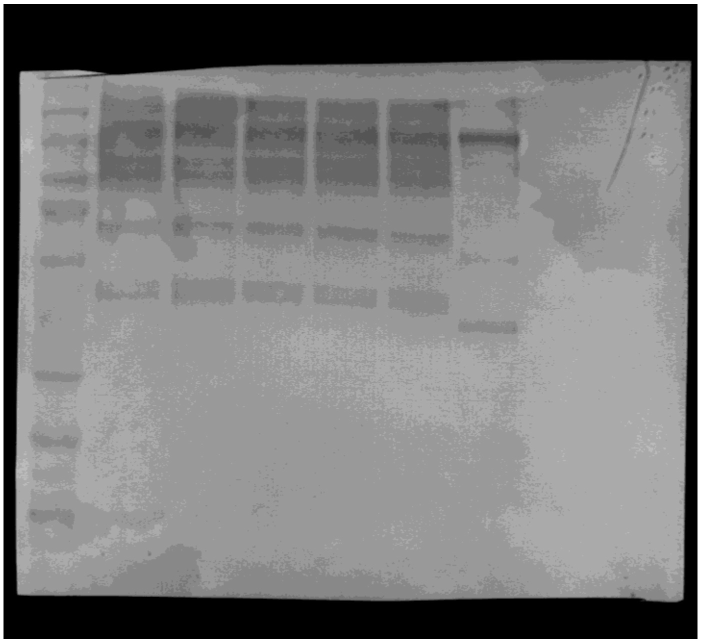

Supplementary Figure S4. Full immunoblot images and colorimetric image of Figure 6.B in the main text. Upper image = Blotted with Anti-APOL3; Lower image = Colorimetric image indicating the ladder. The higher MW bands in the chemiluminescent image depict the cross reactivity of the secondary HRP-mAb with the primary anti-APOL3 antibody used for IP, which was co-eluted with the sample.

Supplementary Figure S5

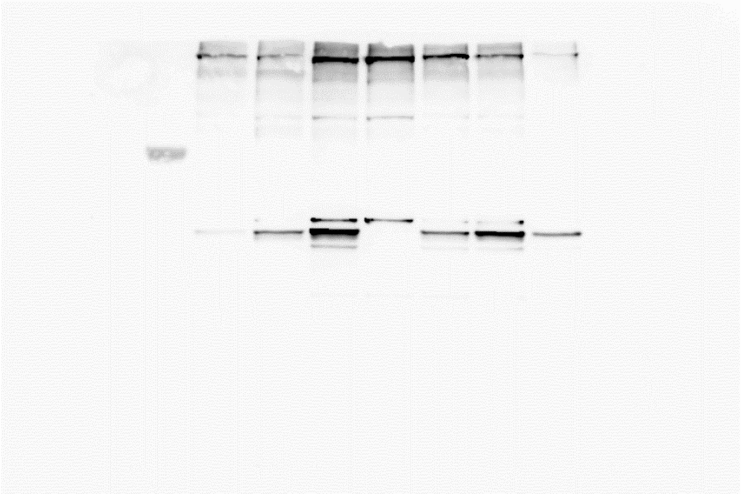

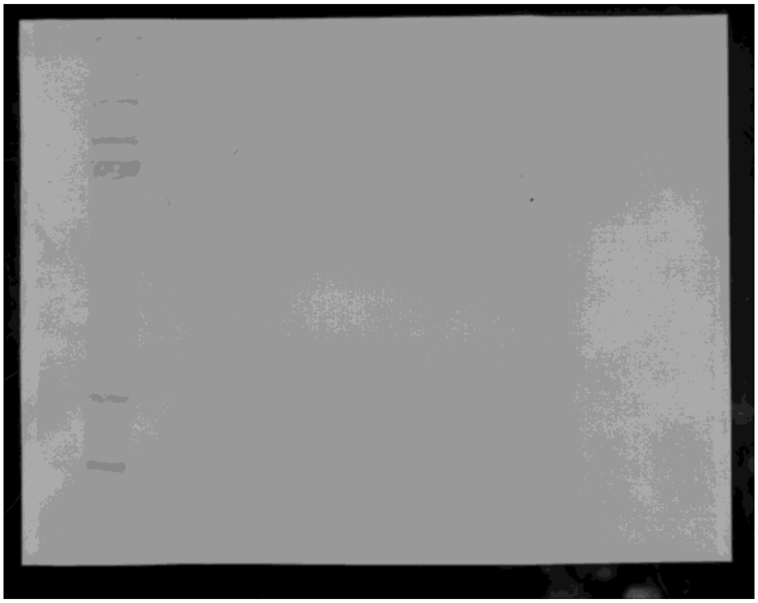

Supplementary Figure S5. Full immunoblot images and colorimetric image of Figure 6.C in the main text. Upper image = Blotted with Anti-APOL1; Lower image = Colorimetric image indicating the ladder.

Supplementary Table S1. Major Trypanosoma Species & Their Impact

Trypanosoma Species	Affected Species	Disease Caused	Region
**T. brucei** (esp. *T. b. gambiense*, *T. b. rhodesiense*)	Humans	African trypanosomiasis (sleeping sickness)	Sub-Saharan Africa
	Wild and domestic animals (e.g., cattle, antelope, pigs)	Animal trypanosomiasis (nagana)	
**T. cruzi**	Humans, dogs, rodents, opossums, armadillos, raccoons	Chagas disease	Central & South America
**T. evansi**	Camels, horses, cattle, dogs, elephants	Surra	Africa, Asia, Latin America
**T. equiperdum**	Horses, donkeys	Dourine (sexually transmitted)	Africa, Asia, South America
**T. vivax**	Cattle, sheep, goats	Animal trypanosomiasis	Africa, Latin America
**T. congolense**	Cattle, goats, sheep, pigs	Animal trypanosomiasis	Sub-Saharan Africa
**T. lewisi**	Rats (occasionally humans)	Usually mild disease	Worldwide (in rodents)
**T. rangeli**	Humans (non-pathogenic), some wild mammals	No significant disease	Central & South America
